# Supplementary material for: Electroinitiated interfacial healing for external pressure-free solid-state sodium metal batteries
Source: Nat Commun. 2025 Oct 30;16:9613. doi: 10.1038/s41467-025-64612-7 (PMC12575646; doi:10.1038/s41467-025-64612-7)
Supplement: Supplementary file 1 — Supplementary Information [file 41467_2025_64612_MOESM1_ESM.pdf]

Supplementary Information

Electroinitiated interfacial healing for external pressure-free solid-state sodium metal batteries

Tingzhou Yang[1,2,3], Siqi Qin[1,3], Shihui Gao[1], Xiaoen Wang[1,*], Dan Luo[1], Yu Shi[2], Qianyi Ma[2], Xinyu Zhang[1,3], Yongguang Zhang[1,*], & Zhongwei Chen[1,2,*]

[1]State Key Laboratory of Catalysis, Dalian Institute of Chemical Physics, Chinese Academy of Sciences, Dalian 116023, China.

[2]Waterloo Institute for Nanotechnology, Department of Chemical Engineering, University of Waterloo, 200 University Ave. W., Waterloo, ON, N2L 3G1, Canada.

[3]School of Chemistry and Chemical Engineering, Nantong University, Nantong 226019, China.

*Correspondence and requests for materials should be addressed to X.W. (email: xiaoenw@dicp.ac.cn), Y.Z. (email: ygzhang@dicp.ac.cn), and Z.C. (email: zwchen@dicp.ac.cn).

List of contents

Supplementary Figures 1–61

Supplementary Tables 1–3

Supplementary References

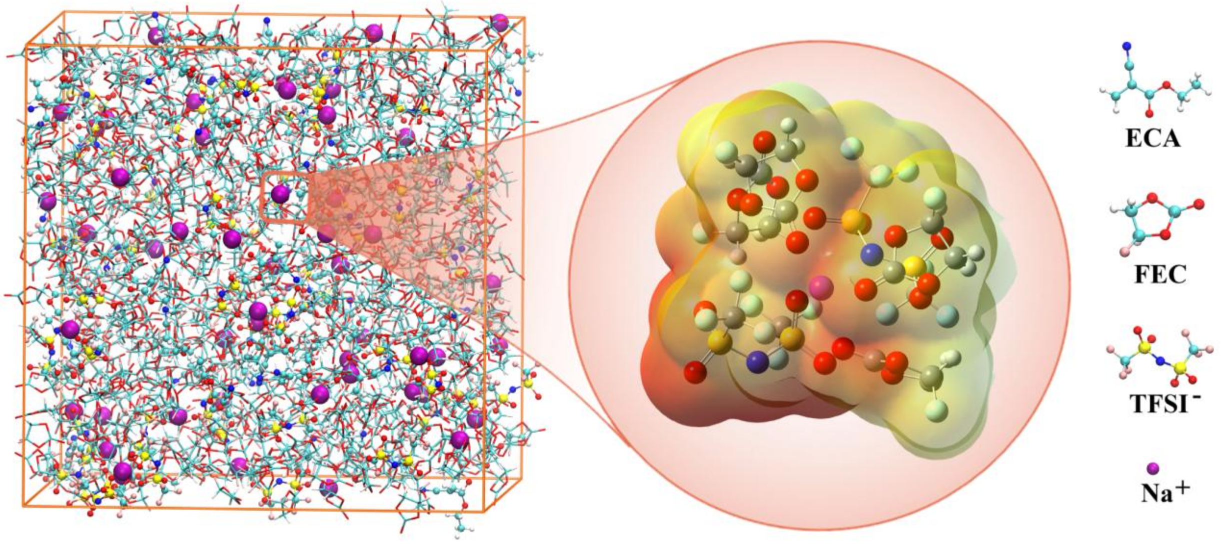

Supplementary Figure 1. Snapshot of molecular dynamics (MD) simulation of 10 vol% Ethyl 2-cyanoacrylate (ECA), and corresponding representative sodium ions (Na ions) coordination structure. MD simulation was first used to screen the optimal formulation of our interfacial mending glue for Na metal SSBs by elucidating its solvation structure and local environment, where the model systems are based on different volume ratios of ECA and Fluoroethylene carbonate (FEC) solvents. As shown in Supplementary Figs. S1–S3, all systems exhibit the first coordination sphere surrounding Na^+ with a maximum peak of radial distribution at 0.238 nm. With the addition of ECA monomers, the coordination number of ECA to Na^+ gradually increases while the coordination number of FEC to Na^+ decreases, suggesting that ECA monomers have participated in the solvation structure of Na^+ and contribute to the performance improvement of our interfacial mending glue used in SSBs (Supplementary Figs. 4 and 5). Then, the Na^+ diffusion coefficient is used to confirm the optimal volume ratio, which can be calculated from the long-time limit of the mean square displacement according to the Einstein diffusion equation. It is worth noting that the higher the addition amount is not the better, the volume ratio between ECA and FEC of 3:7 shows the highest diffusion coefficient of 6.69×10^{-7} cm^{-2} s^{-1} compared with the volume ratios of 1:9 (4.33×10^{-7} cm^{-2} s^{-1}) and 5:5 (3.61×10^{-7} cm^{-2} s^{-1}, Supplementary Fig. 6).

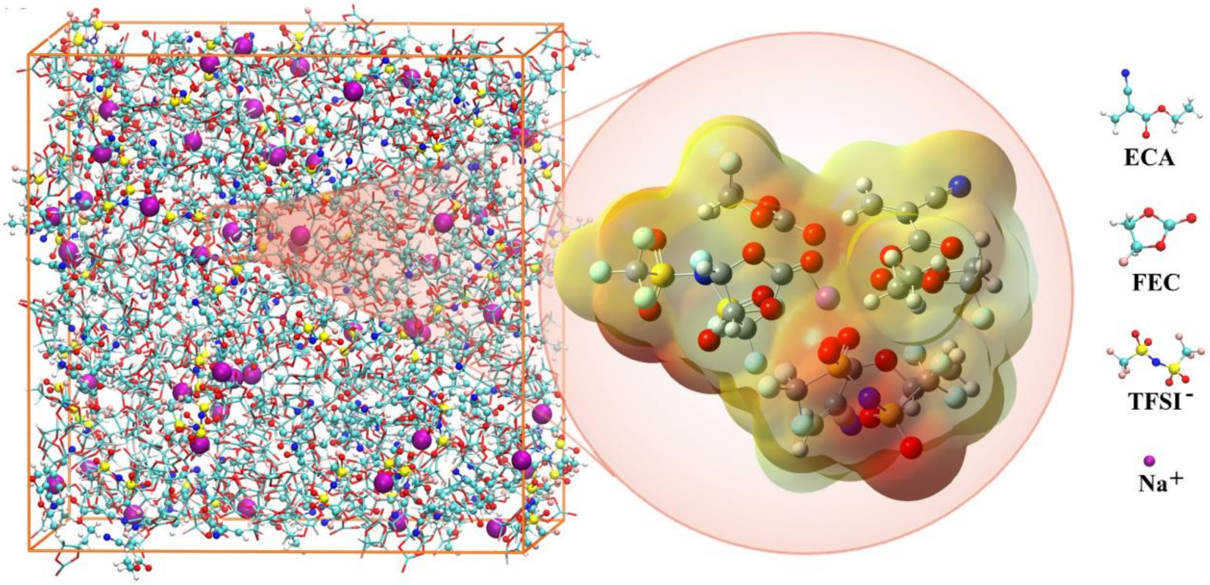

Supplementary Figure 2. Snapshot of MD simulation of 30 vol% ECA, and corresponding representative Na^+ coordination structure.

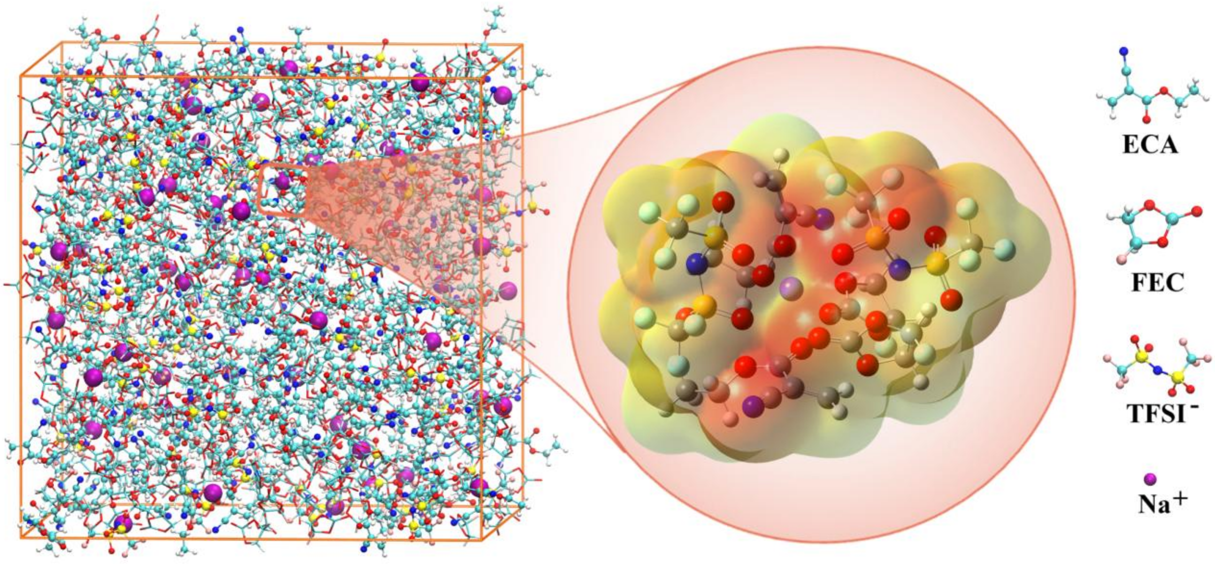

Supplementary Figure 3. Snapshot of MD simulation of 50 vol% ECA, and corresponding representative Na^+ coordination structure.

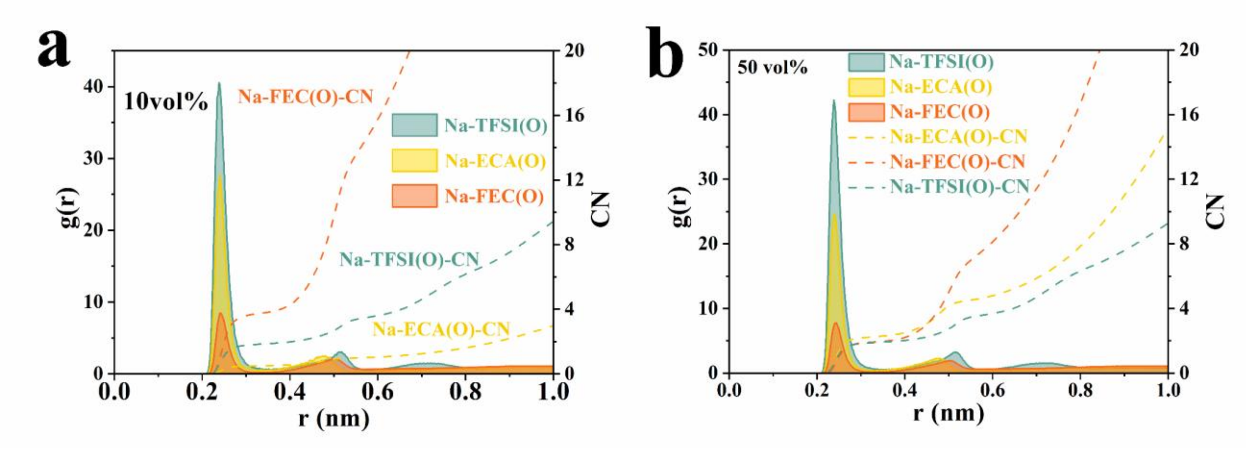

Supplementary Figure 4. Radial distribution functions at the equilibrium of **a** 10 vol% and **b** 50 vol% ECA.

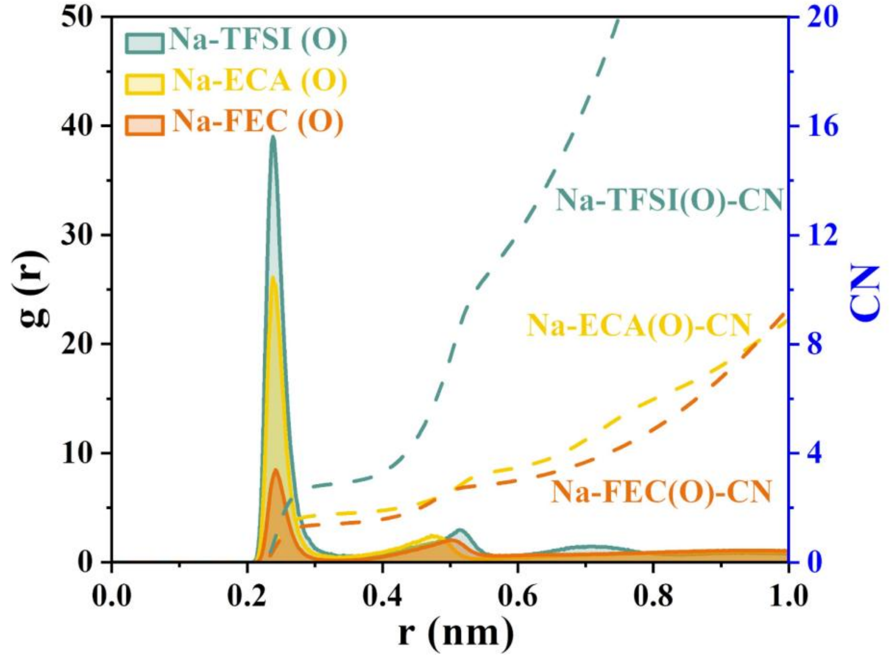

Supplementary Figure 5. Radial distribution functions at an equilibrium of 30 vol% ECA.

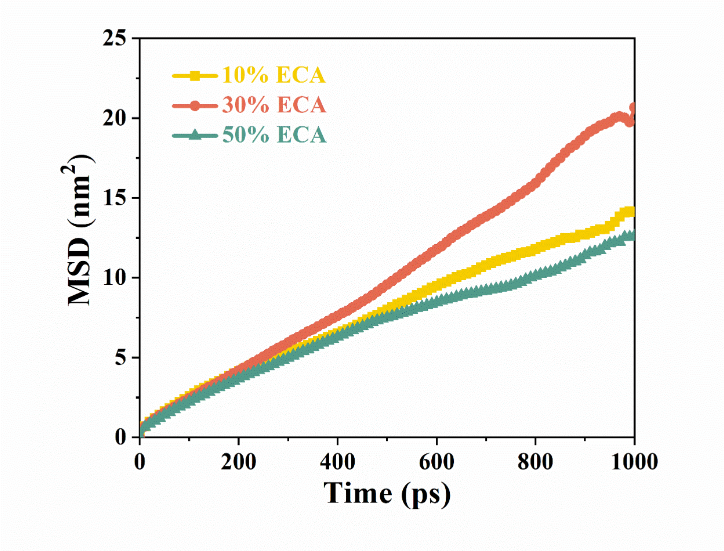

Supplementary Figure 6. Mean square displacement of Na^+ with different radios of ECA.

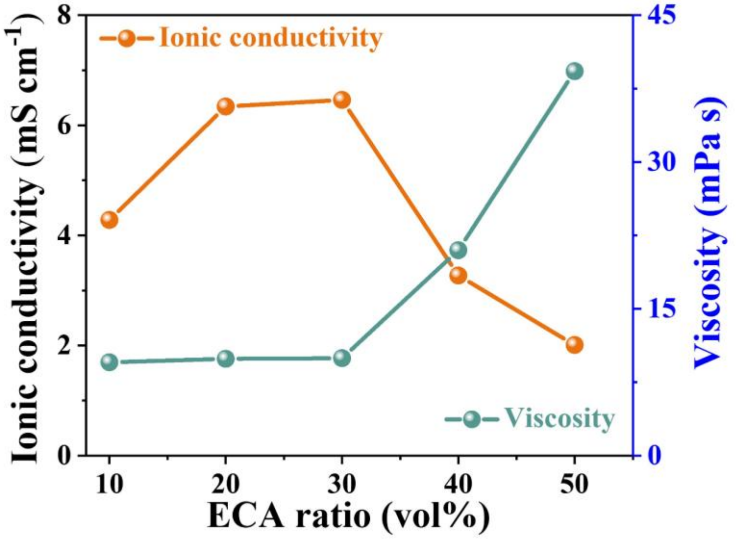

Supplementary Figure 7. Average ionic conductivity and viscosity of interfacial mending glue with different volume ratios. The ionic conductivity increases from 4.28 to 6.46 mS cm^{-1} and the viscosity is maintained at a small value of around 9.96 mPa s as the proportion of ECA increases from 10 vol% to 30 vol%, which competes with conventional liquid electrolytes. After the proportion exceeds 30 vol%, the ionic conductivity gradually decreases and the viscosity significantly increases, further proving the accuracy of the preliminary screening by computer simulations.

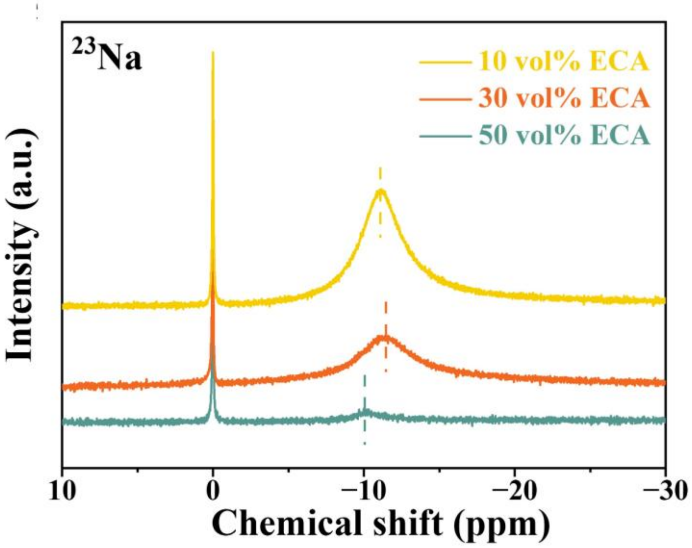

Supplementary Figure 8. ^{23}Na nuclear magnetic resonance (NMR) spectra of interfacial mending glue with different volume ratios. The corresponding ^{23}Na NMR spectrum exhibits a downfield shift for the optimal formula compared with other formulas, indicating the weakened interaction between cations and anions. These phenomena are consistent with MD simulations, where the addition of ECA can promote the interaction between oxygen atoms and Na^+, thus contributing to the increase of ionic conductivity.

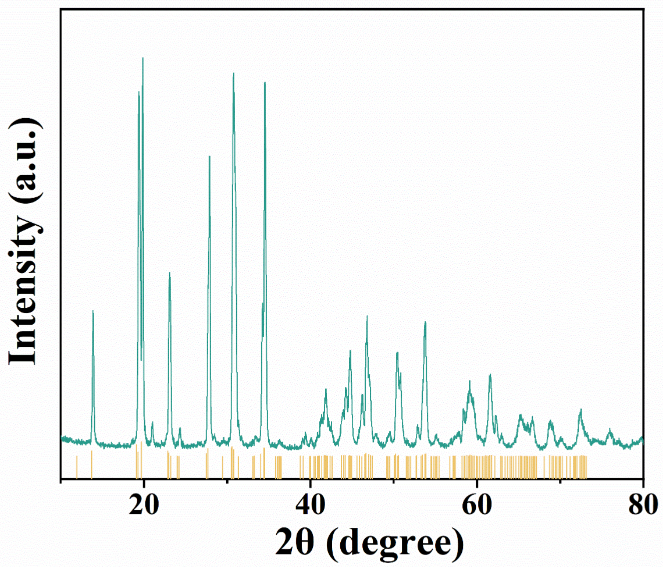

Supplementary Figure 9. X-ray diffraction (XRD) curves of the $Na_{3.4}Zr_{1.9}Zn_{0.1}Si_{2.2}P_{0.8}O_{12}$ (NZZSPO) solid-state electrolyte.

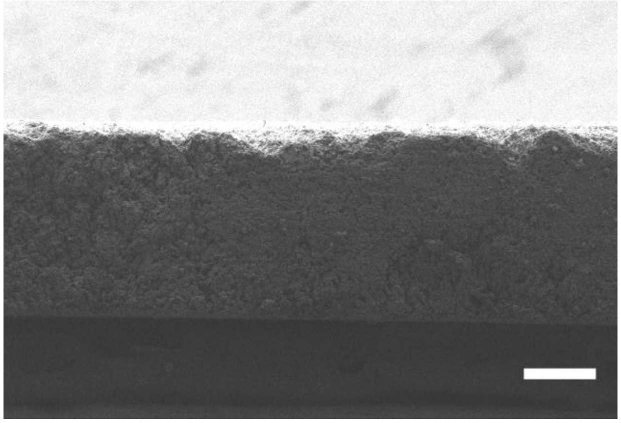

Supplementary Figure 10. SEM image of the $Na_{3.4}Zr_{1.9}Zn_{0.1}Si_{2.2}P_{0.8}O_{12}$ solid-state electrolyte after pressing into pieces. Scale bar, 50 μm.

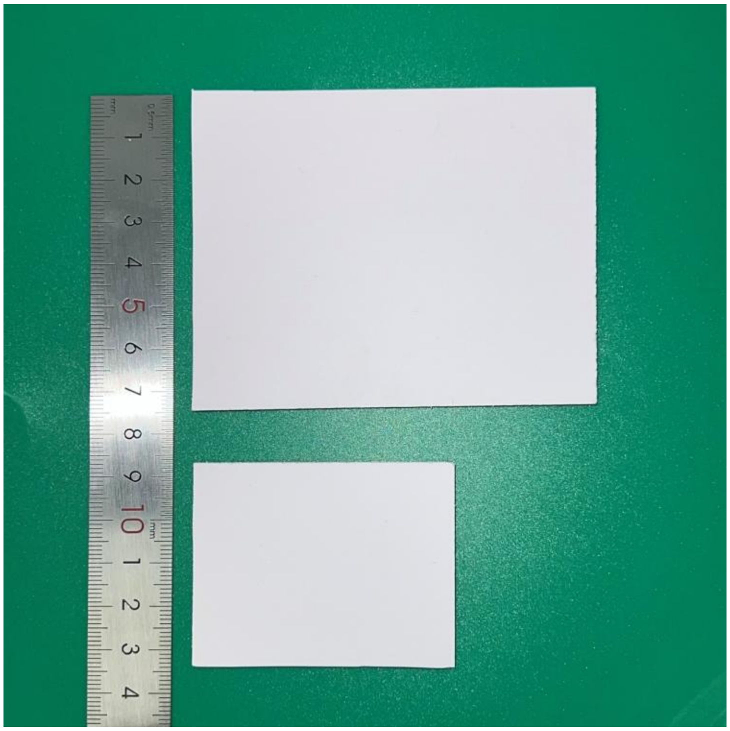

Supplementary Figure 11. Photograph of the obtained $Na_{3.4}Zr_{1.9}Zn_{0.1}Si_{2.2}P_{0.8}O_{12}$ solid-state electrolyte with different sizes, which can be used in different types of solid-state Na metal pouch cells.

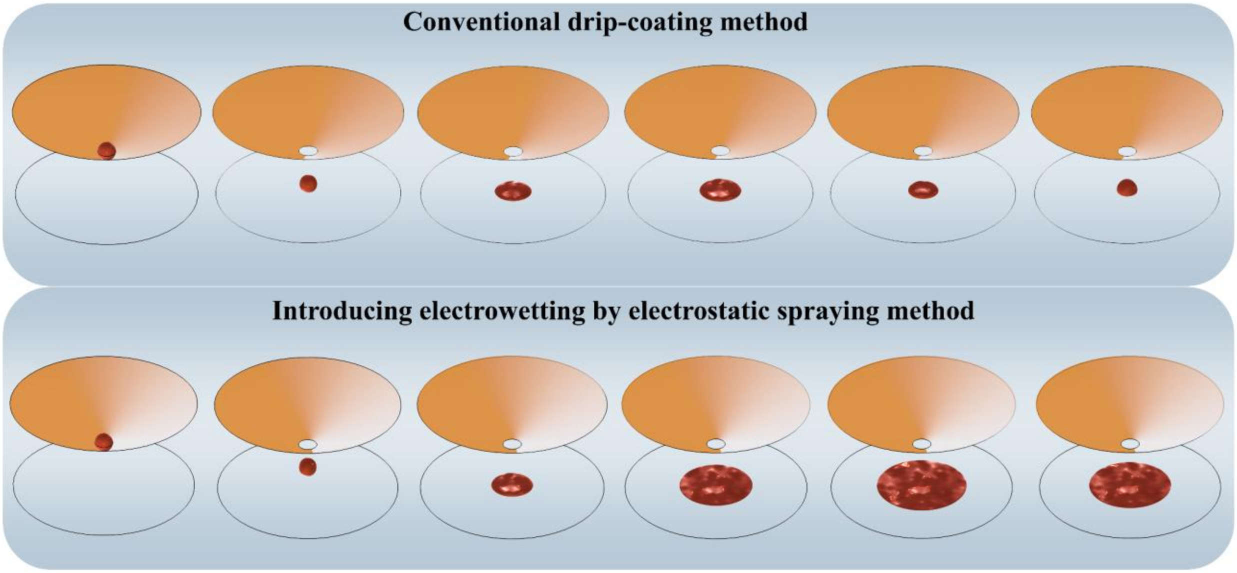

Supplementary Figure 12. Simulated coating process of IMG microdroplets through conventional drip-coating method (top) and electroinitiated accelerated polymerization (EAP) healing strategy (bottom).

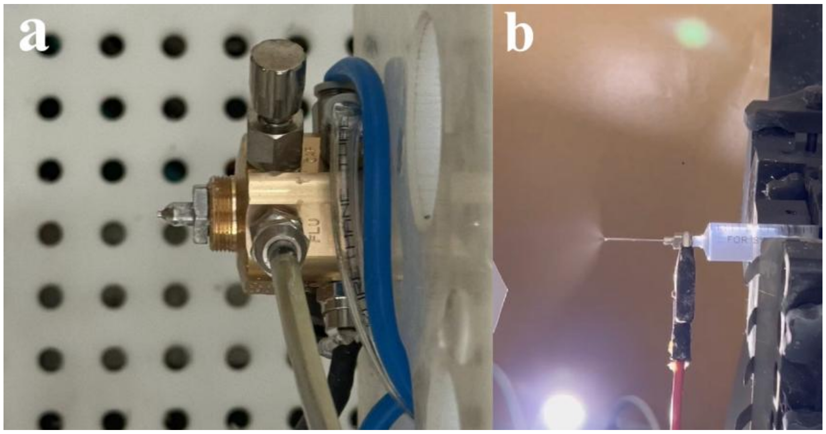

Supplementary Figure 13. Photograph of designed nozzle for the improved electrostatic spraying process of interfacial mending glue (IMG) for **a** pouch cell and **b** coin cell.

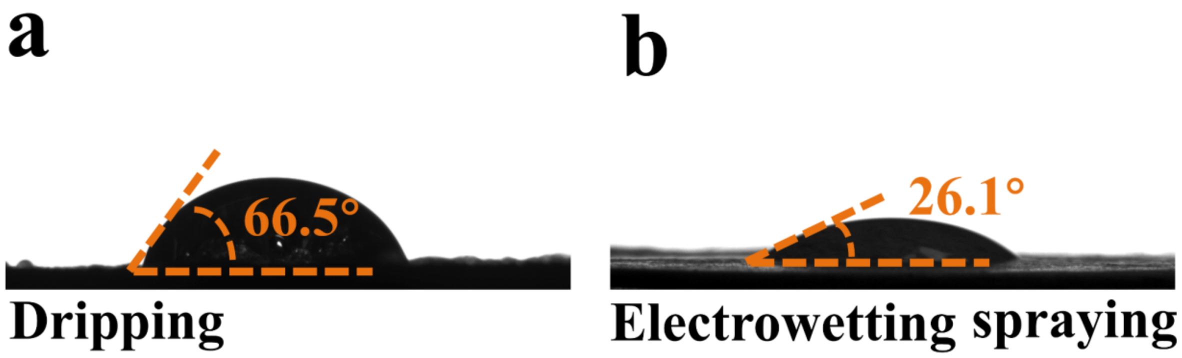

Supplementary Figure 14. Contact angles between Na metal and IMG microdroplets with **a** conventional drip-coating method and **b** EAP strategy.

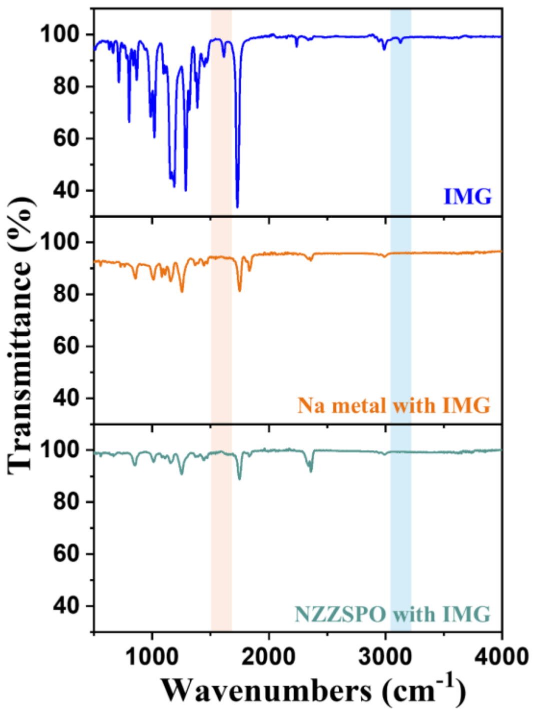

Supplementary Figure 15. Fourier-transform infrared (FT-IR) spectrums of various samples operated in the glove box with a relative humidity of less than 0.01 ppm, where IMG microdrops are sprayed onto the surface of Na metal and NZZSPO under a higher applied electric field. Electrons can attack the β-unsaturated carbon in the ECA, forming a carbon anion, which further attacks other ECA monomers and induces the continuous polymerization reaction into long and strong chains.

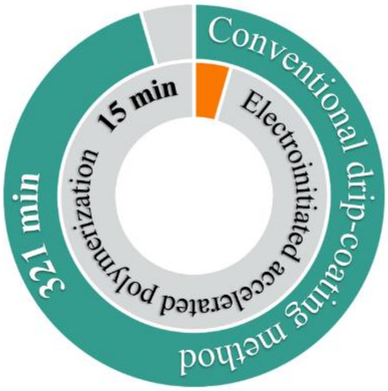

Supplementary Figure 16. Comparison of polymerization time for various strategies, where EAP strategy speeds up by 21.4 times.

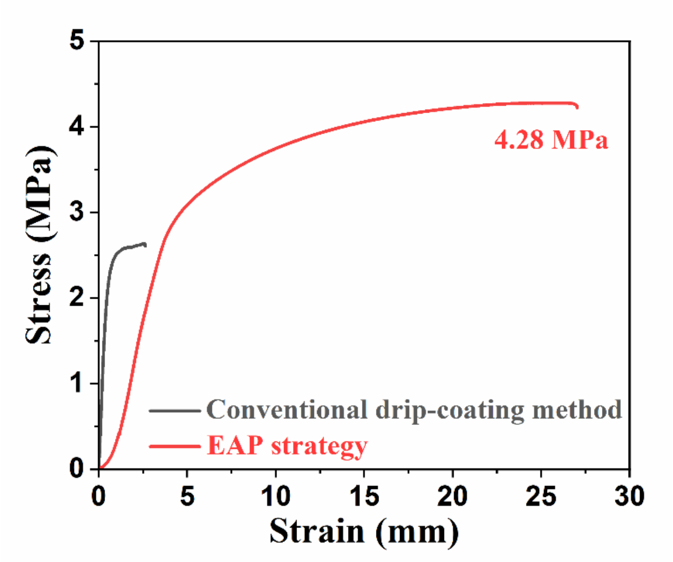

Supplementary Figure 17. Stress-strain curves of EAP strategy and conventional drip-coating method measured by electronic universal testing machines.

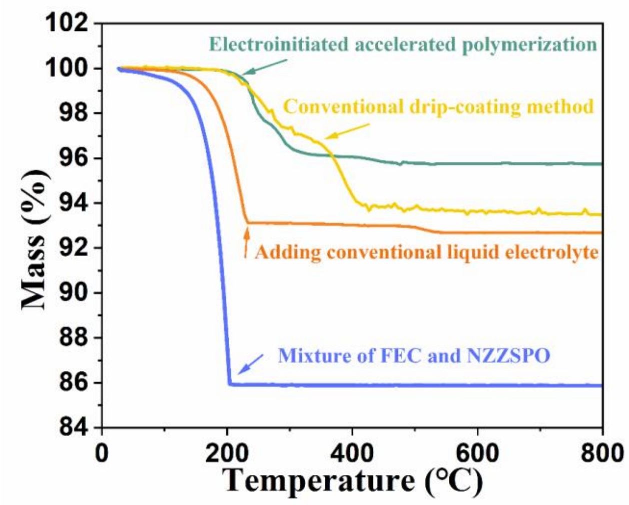

Supplementary Figure 18. Thermogravimetric analysis curves of NZZSPO with interfacial optimization strategy including adding conventional liquid electrolyte, conventional drip-coating method, and electroinitiated accelerated polymerization strategy.

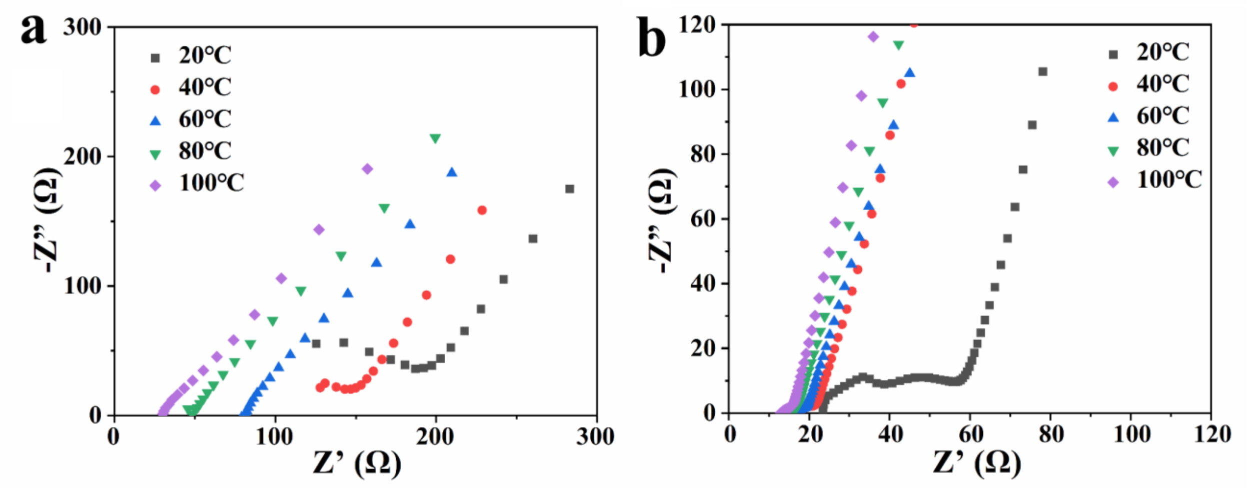

Supplementary Figure 19. Nyquist plots of the solid-state cells with ceramic electrolytes and Au-blocking electrodes modified by adding **a** conventional liquid electrolytes and **b** IMG using EAP strategy measured at different temperatures.

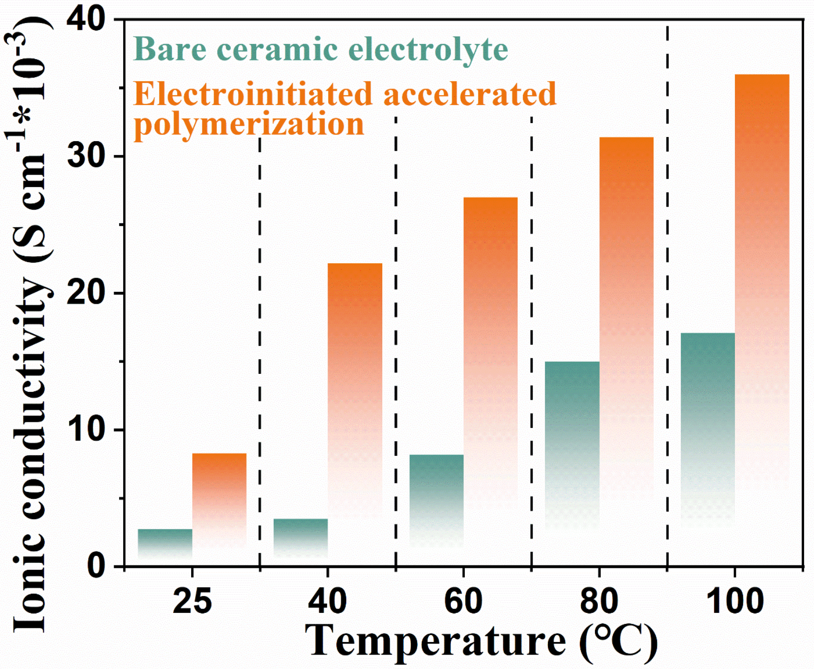

Supplementary Figure 20. Ionic conductivities of various strategies under various temperatures.

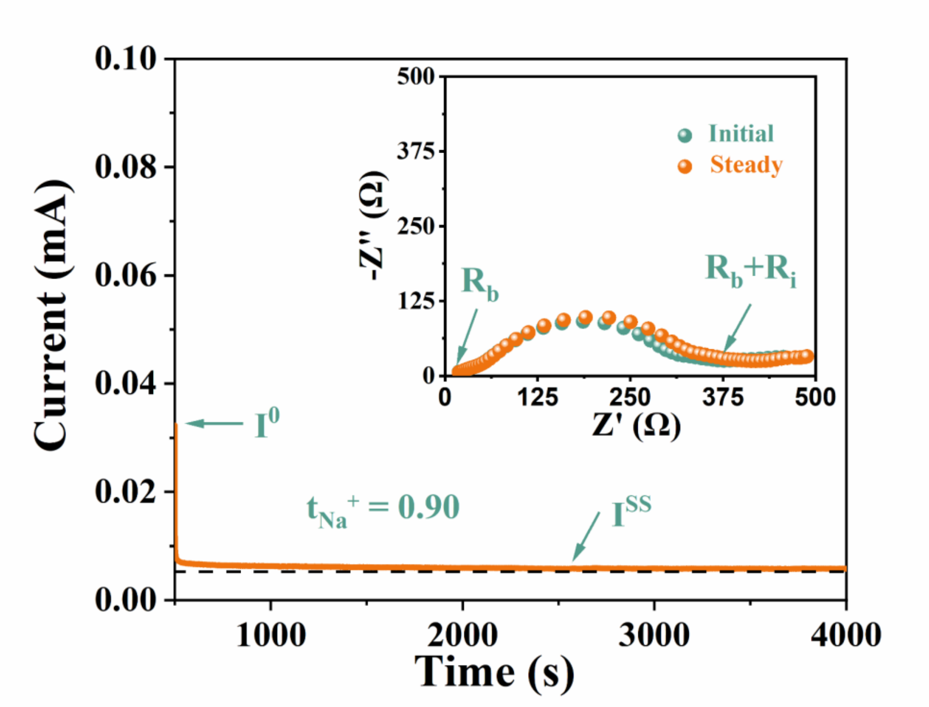

Supplementary Figure 21. Chronoamperometry profile of symmetric cell optimized by EAP strategy under a polarization voltage of 10 mV and corresponding EIS spectra (inset).

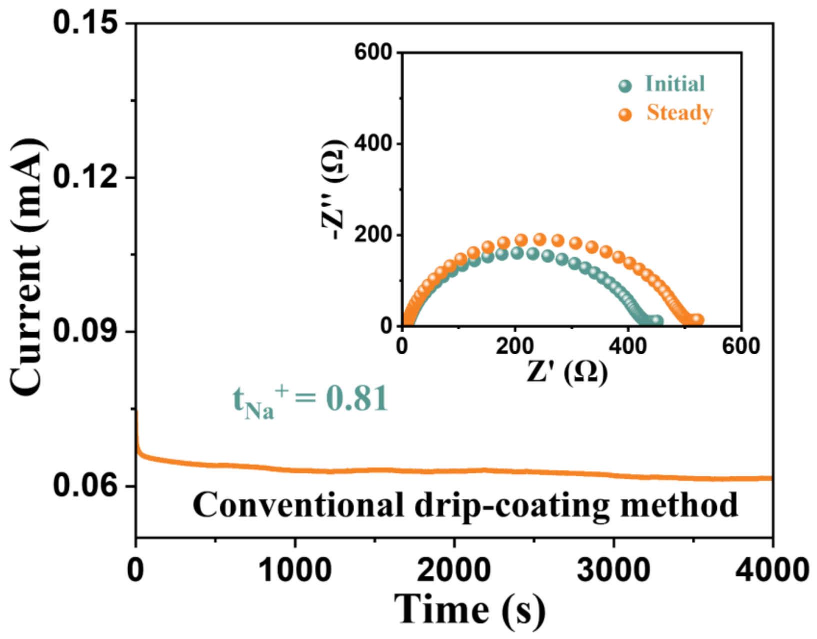

Supplementary Figure 22. Chronoamperometry profile of symmetric cell optimized by conventional drip-coating method and corresponding EIS spectra (inset).

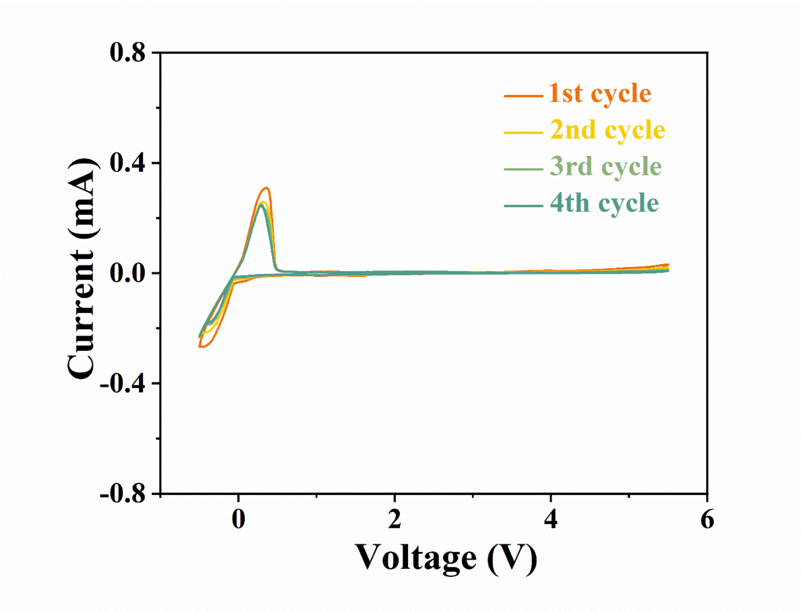

Supplementary Figure 23. The CV curves of oxide solid electrolytes optimized by EAP strategy at a scan rate of 1.0 mV s^{-1}.

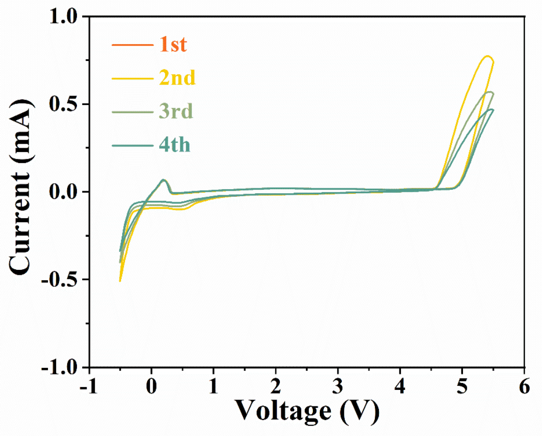

Supplementary Figure 24. The CV curves of conventional liquid electrolytes between −0.5 and 5.5 V at a scan rate of 1.0 mV s^{-1}.

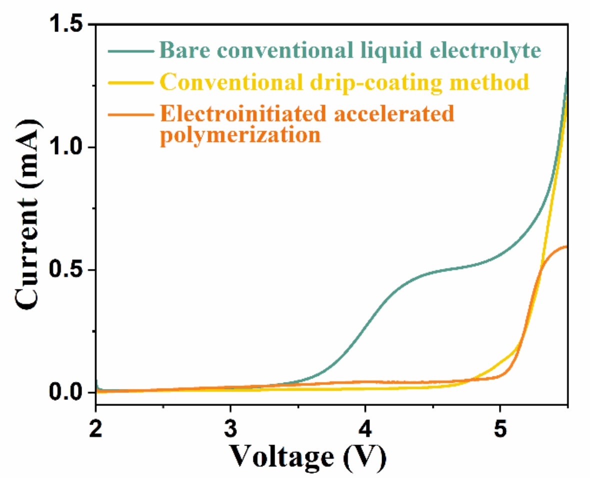

Supplementary Figure 25. LSV curves of bare conventional liquid electrolyte and oxide solid electrolytes optimized by conventional drip-coating method and EAP strategy.

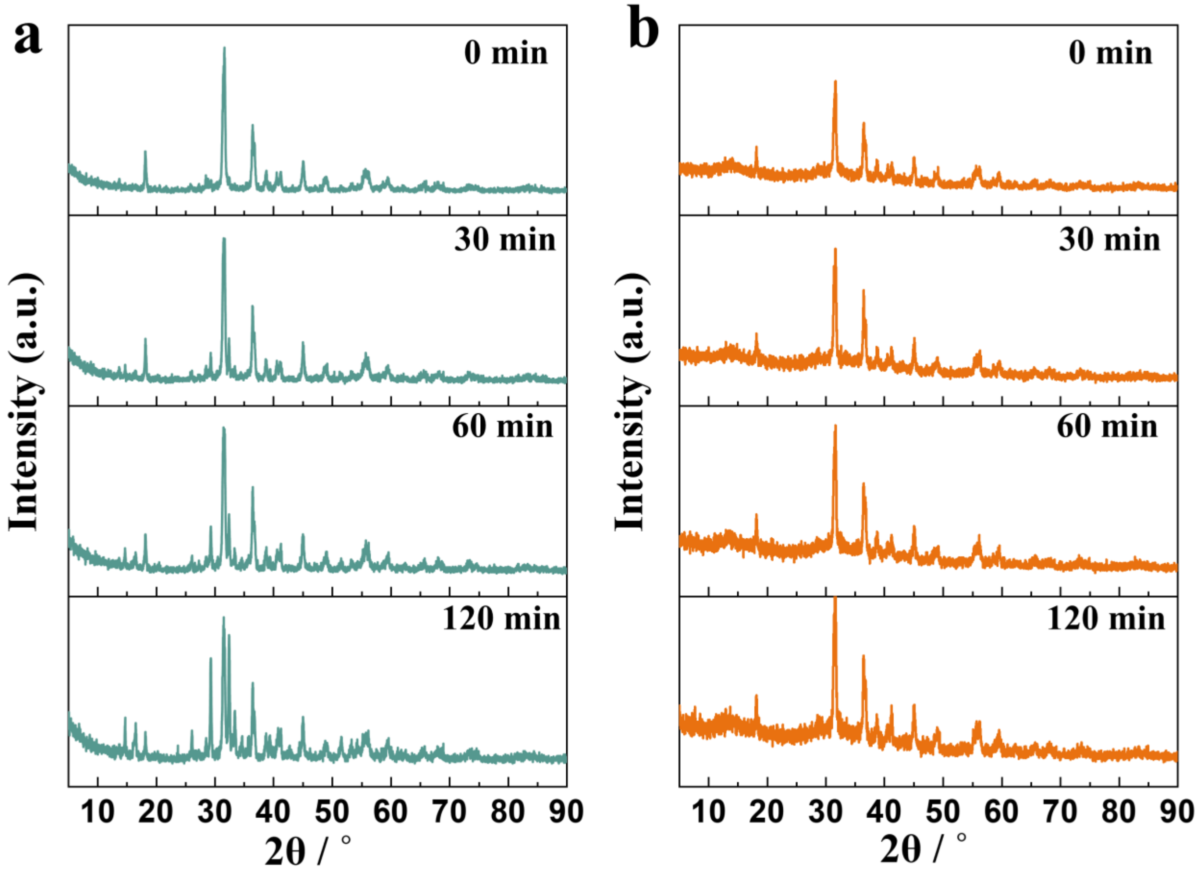

Supplementary Figure 26. XRD curves of Na-based sulfide solid electrolytes (NaPSCl) **a** without and **b** with our polymerized IMG healing layer after exposure to ambient air. Compared with pure NaPSCl with a significant change, XRD curves after covering our polymerized IMG healing layer using the EAP strategy are consistent with the new NaPSCl sheet without any change.

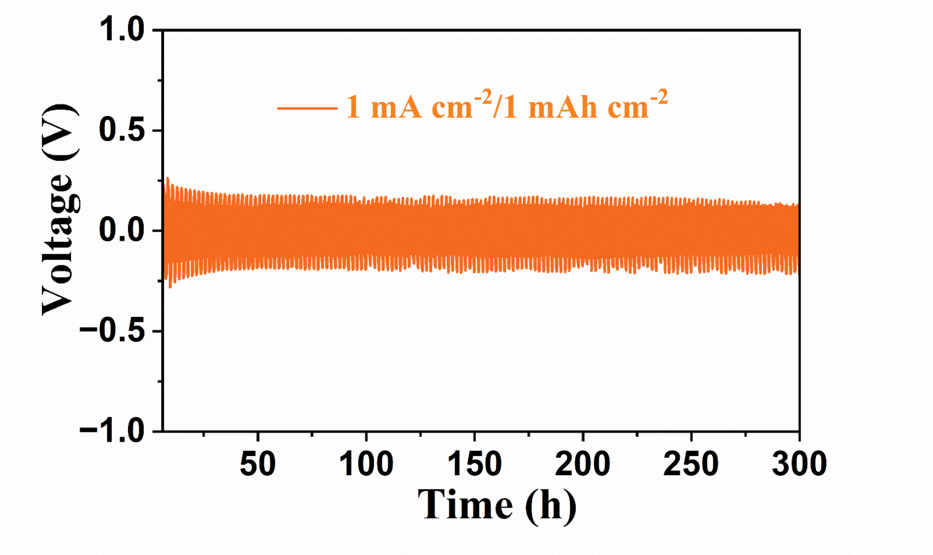

Supplementary Figure 27. Voltage-time profiles of the Na metal-based solid-state symmetric cell with our EAP strategy at the current density of 1.0 mA cm^{-2} with an area capacity of 1.0 mAh cm^{-2}.

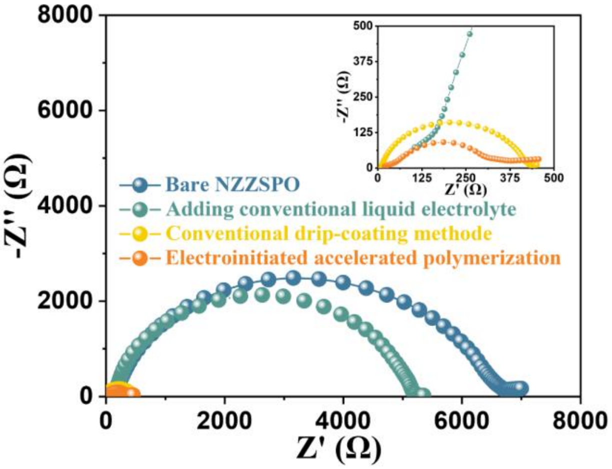

Supplementary Figure 28. Nyquist plots of the symmetric Na metal solid-state cell optimized by EAP strategy, conventional drip-coating method, and conventional liquid electrolyte. The inset shows a zoomed-in view of the high-frequency region.

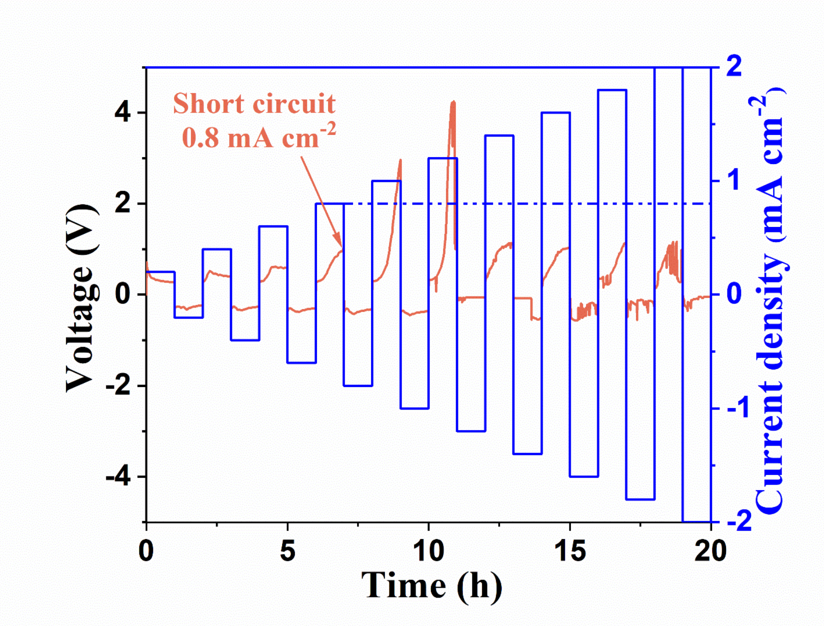

Supplementary Figure 29. Na platting/stripping performance of NZZSPO electrolyte with conventional liquid electrolyte in Na metal symmetric cell at different current densities, where the short-circuit signal with a significant increase in overpotential can be observed after the current exceeded 0.8 mA cm^{-2}.

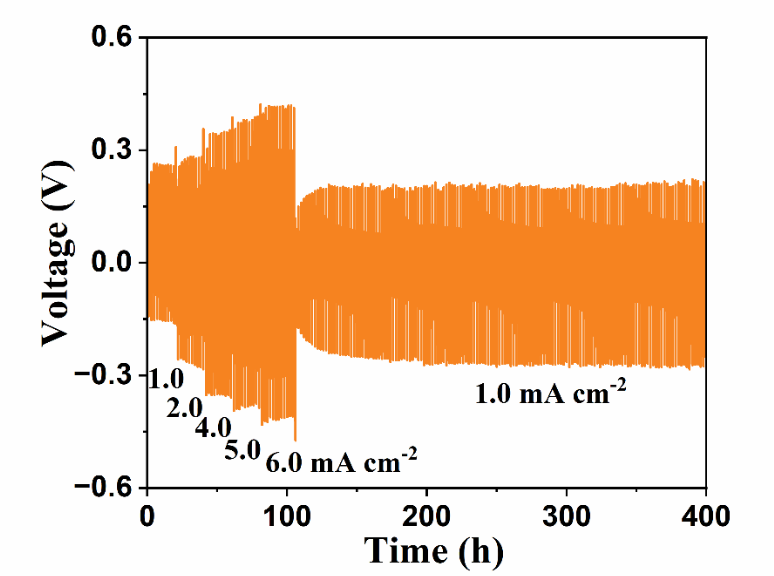

Supplementary Figure 30. Rate capability under various current densities from 1.0 to 6.0 mA cm^{-2}.

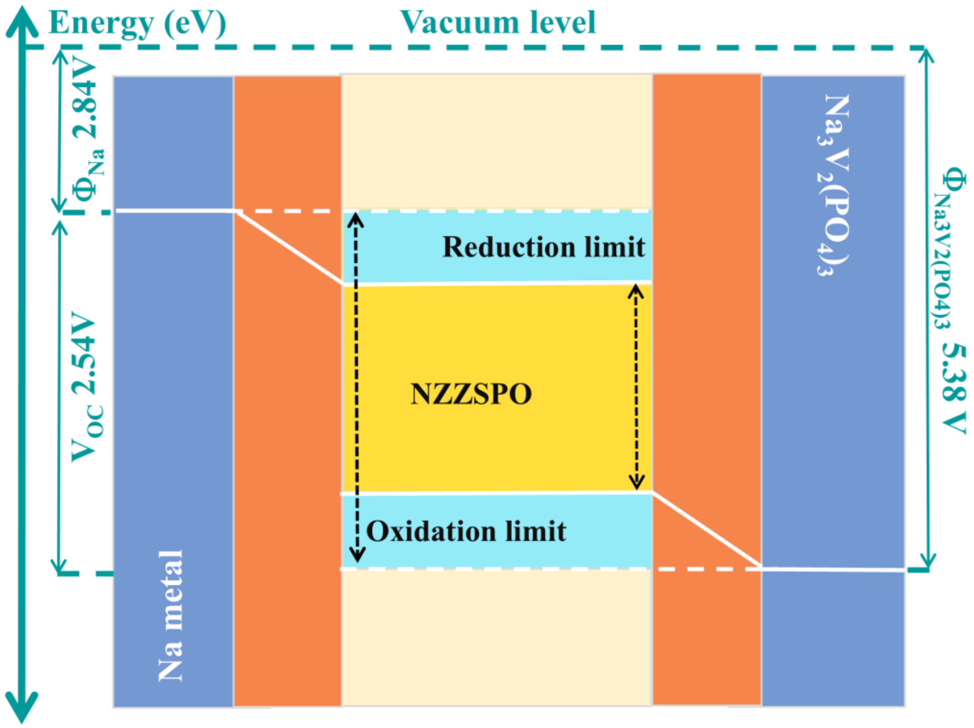

Supplementary Figure 31. Schematic illustration of open-circuit energy diagram for the SSB system based on DFT results.

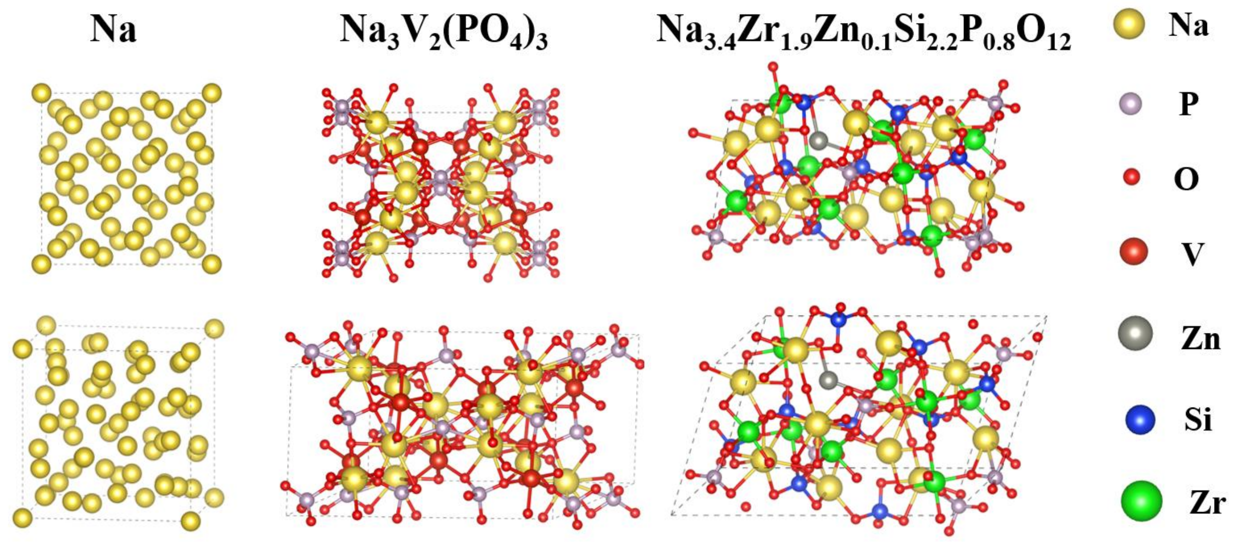

Supplementary Figure 32. Molecular structures of Na, $Na_3V_2(PO_4)_3$, and $Na_{3.4}Zr_{1.9}Zn_{0.1}Si_{2.2}P_{0.8}O_{12}$.

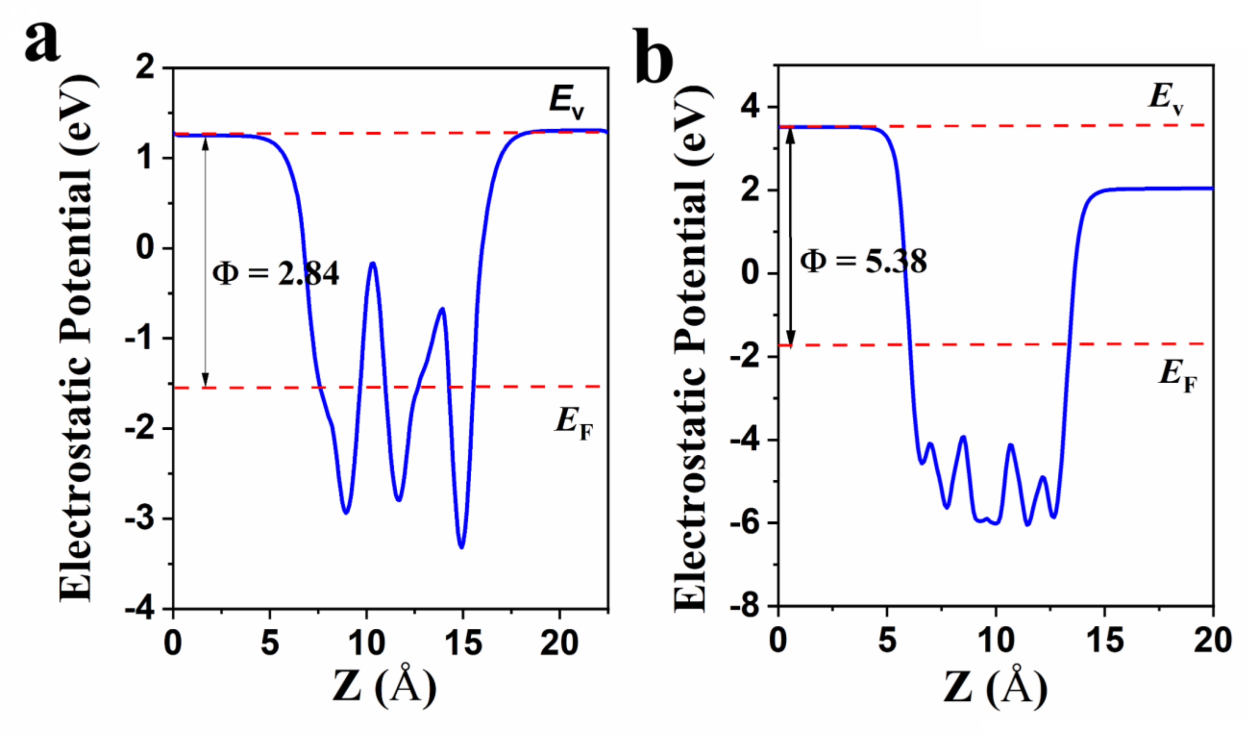

Supplementary Figure 33. The chemical potentials of **a** Na and **b** $Na_3V_2(PO_4)_3$ positive electrode.

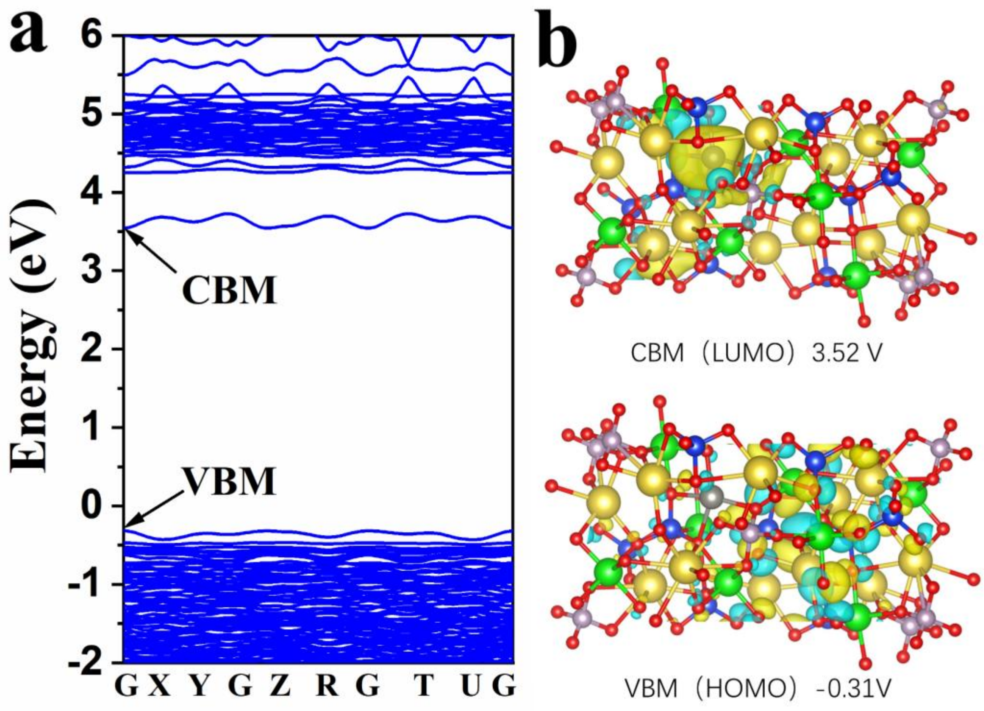

Supplementary Figure 34. a Calculated band dispersion of $Na_{3.4}Zr_{1.9}Zn_{0.1}Si_{2.2}P_{0.8}O_{12}$ and potentials lie between conduction band minimum (CBM) and valence band maximum (VBM). **b** HOMO-LUMO orbital and gap diagrams of $Na_{3.4}Zr_{1.9}Zn_{0.1}Si_{2.2}P_{0.8}O_{12}$, where the Na, Zr, Zn, Si, P, and O atoms are represented by yellow, green, grey, blue, light pink, and red spheres, respectively.

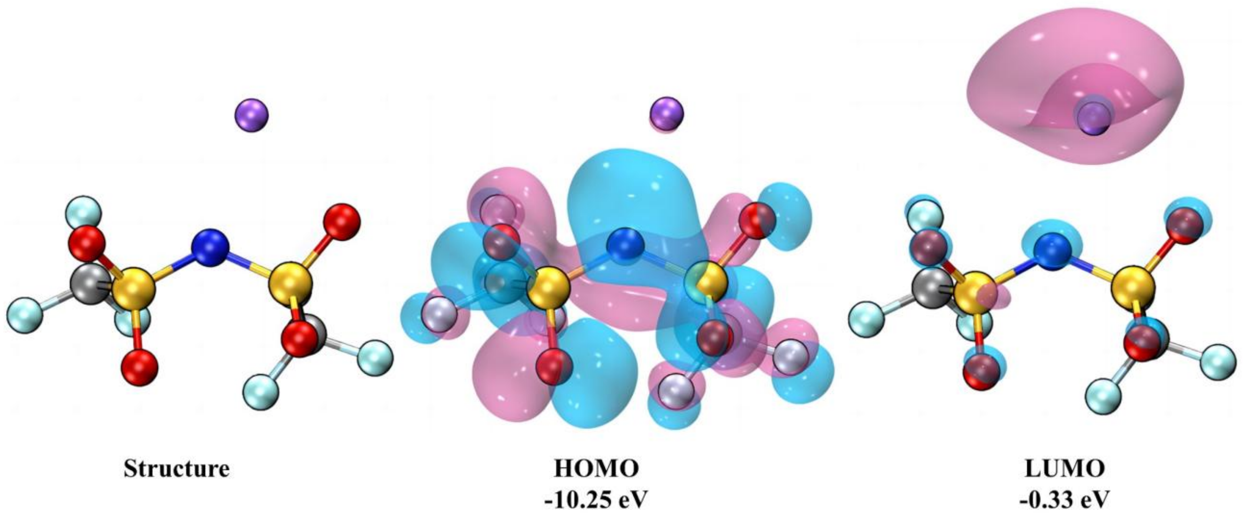

Supplementary Figure 35. HOMO-LUMO orbital and gap diagrams of NaTFSI, where the Na, S, N, C, O, and F atoms are represented by purple, yellow, blue, grey, red, and light blue spheres, respectively.

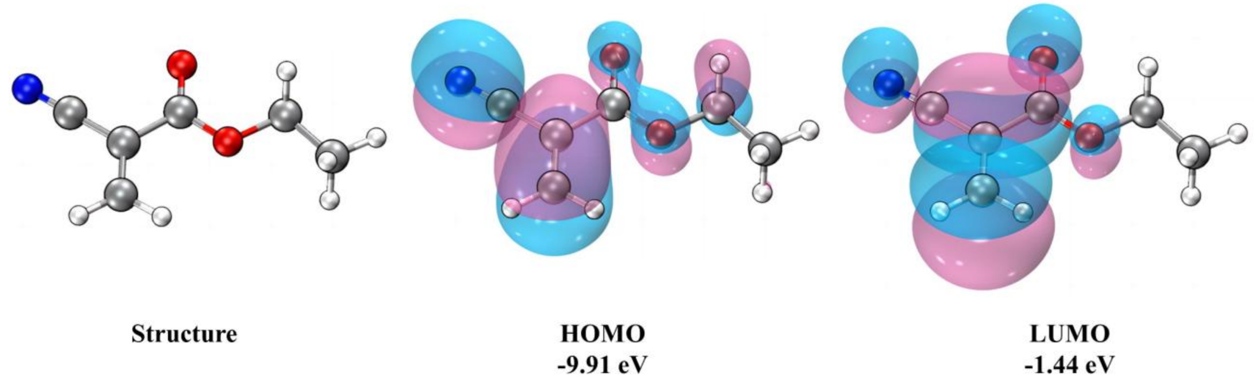

Supplementary Figure 36. HOMO-LUMO orbital and gap diagrams of ECA monomer, where the C, N, O, and H atoms are represented by grey, blue, red, and white spheres, respectively.

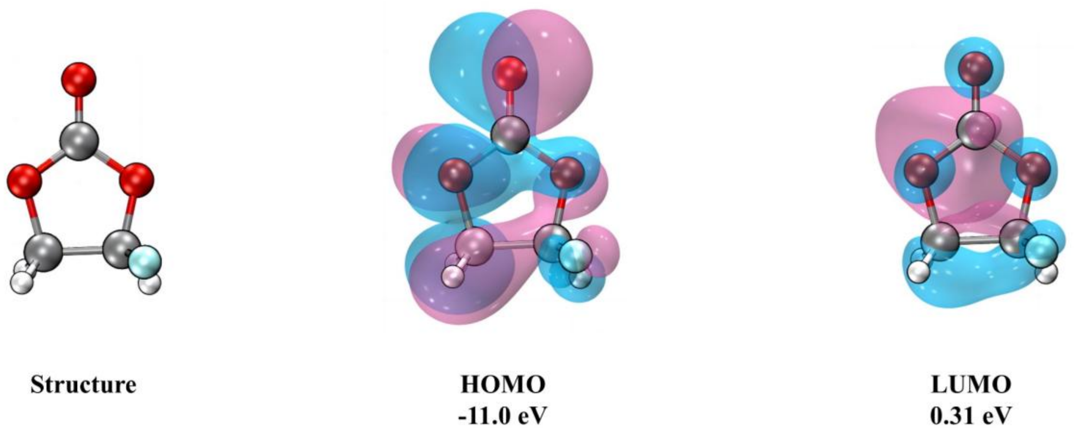

Supplementary Figure 37. HOMO-LUMO orbital and gap diagrams of FEC, where the C, O, H, and F atoms are represented by grey, red, white, and light blue spheres, respectively.

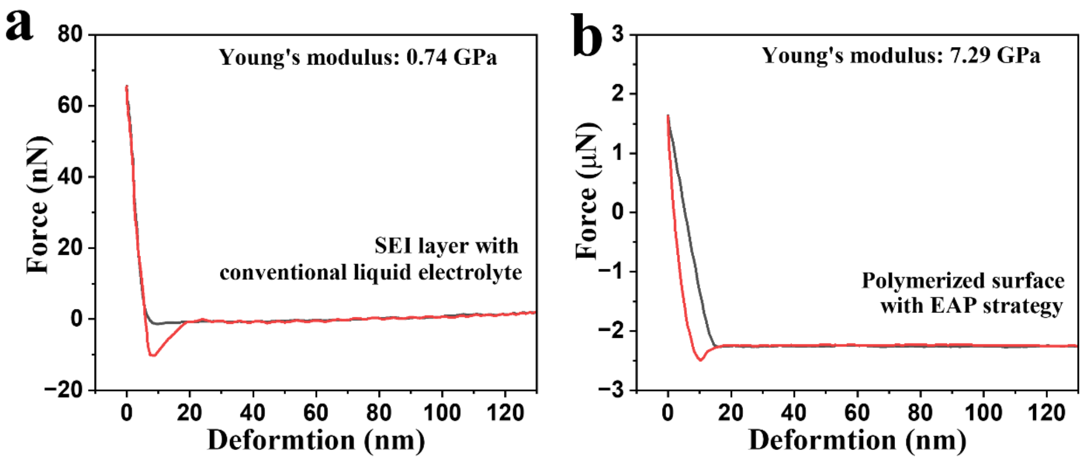

Supplementary Figure 38. Indentation curves of **a** the SEI layer on Na metal negative electrode after adding conventional liquid electrolyte (CLE) and **b** the surface of polymerized IMG formed via the EAP strategy enabled by atomic force microscopy analysis.

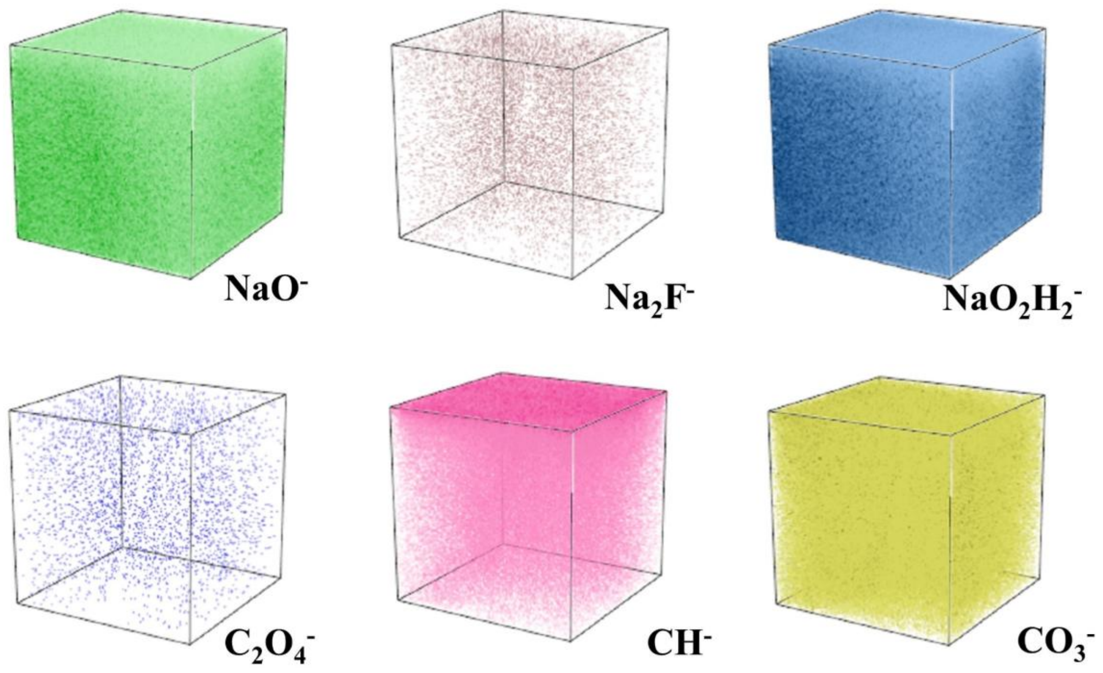

Supplementary Figure 39. TOF-SIMS 3D reconstruction of the representative inorganic signals (NaO^-, Na_2F^-, and $NaO_2H_2^-$) and organic signals ($C_2O_4^-$, CH^-, and CO_3^-) on the Na metal surface after adding CLE.

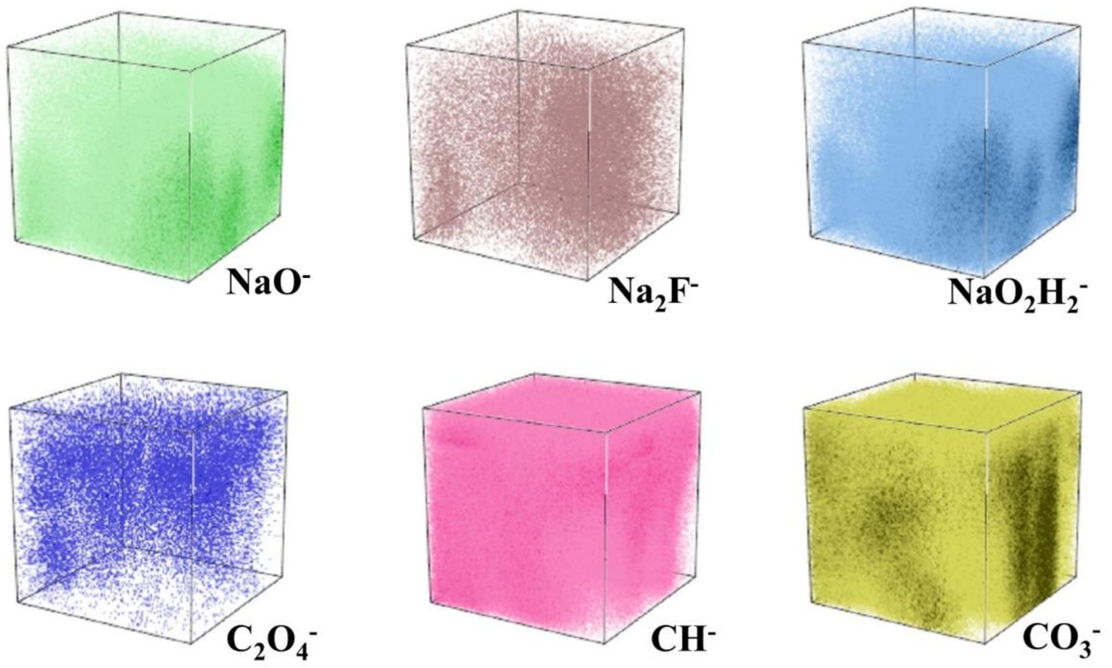

Supplementary Figure 40. TOF-SIMS 3D reconstruction of the representative inorganic signals (NaO^-, Na_2F^-, and $NaO_2H_2^-$) and organic signals ($C_2O_4^-$, CH^-, and CO_3^-) on the Na metal surface after using the EAP strategy.

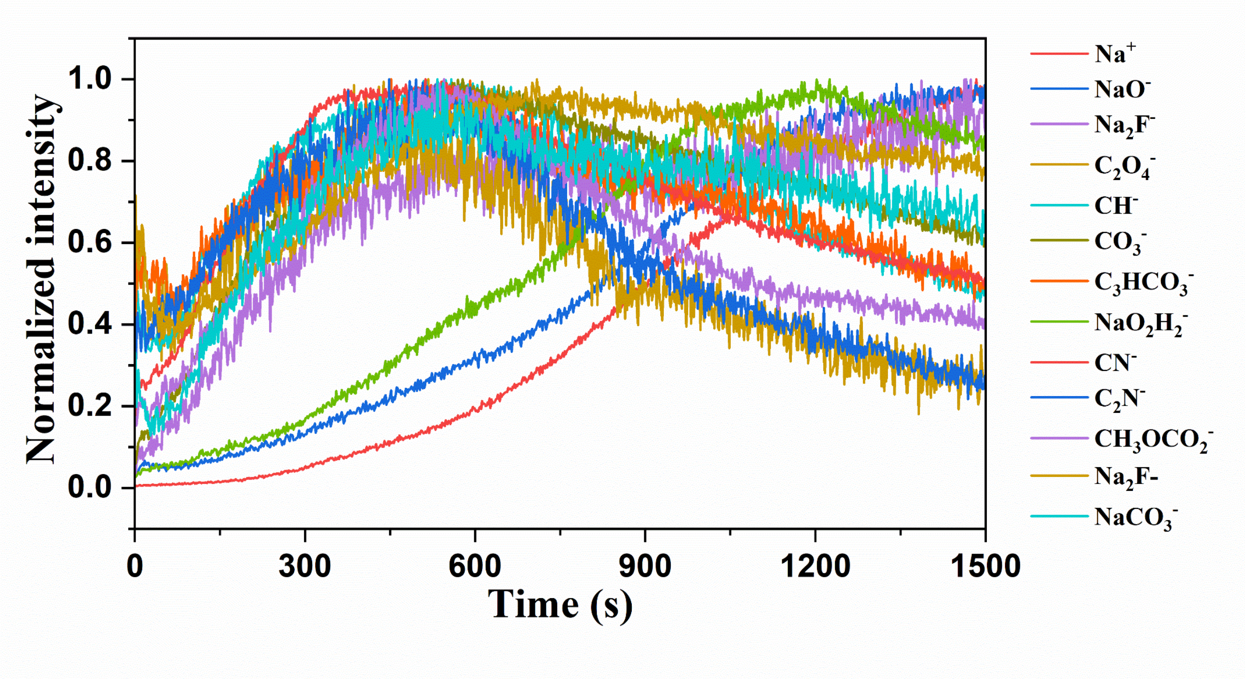

Supplementary Figure 41. TOF-SIMS curves of the representative species of SEI layers formed at the surface of Na metal negative electrode in SSBs after using the EAP strategy.

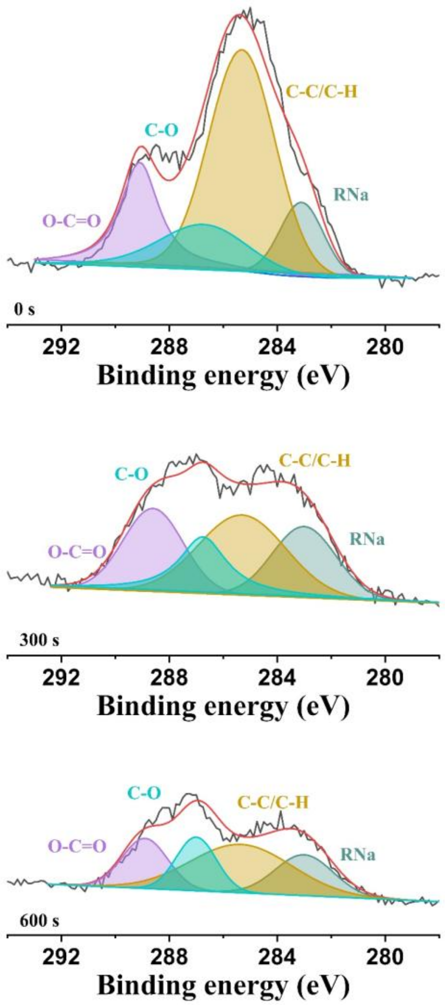

Supplementary Figure 42. The surface and depth profiles XPS analyzation of C 1*s* for Na metal negative electrode after adding CLE.

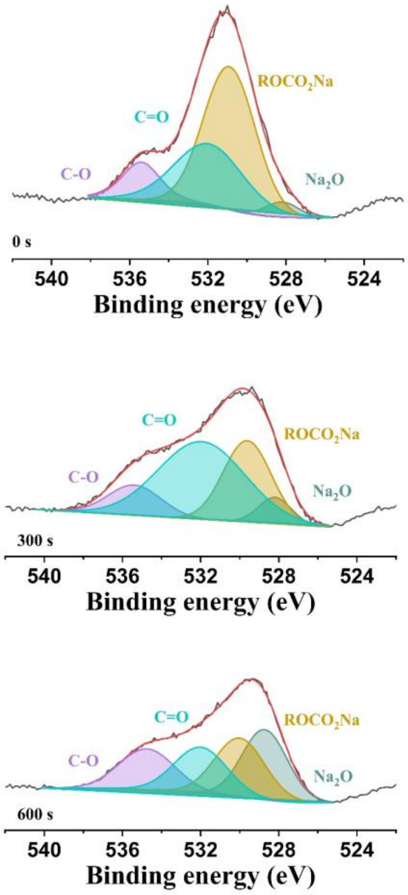

Supplementary Figure 43. The surface and depth profiles XPS analyzation of O 1*s* for Na metal negative electrode after adding CLE.

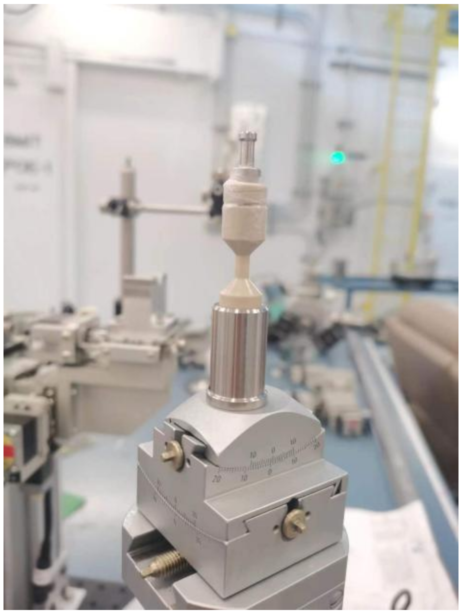

Supplementary Figure 44. The homemade cell for the measurement of Nano-CT.

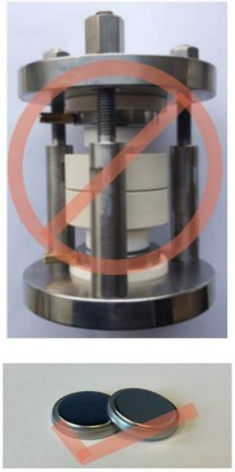

Supplementary Figure 45. Owing to the interfacial optimization strategy of our EAP strategy, all results are measured in coin cells without any clamping force, the same as conventional coin cells.

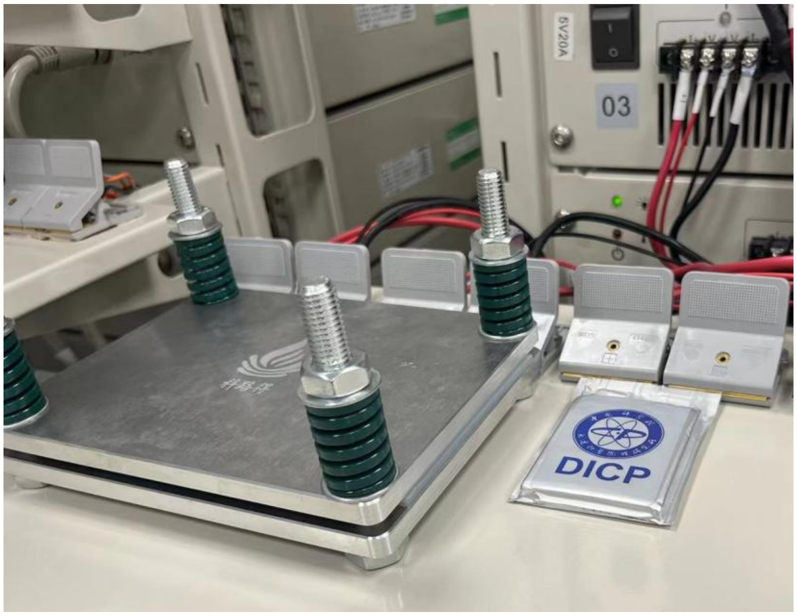

Supplementary Figure 46. Owing to the interfacial optimization strategy of our EAP strategy, all results are measured in pouch cells without using any high-pressure modules.

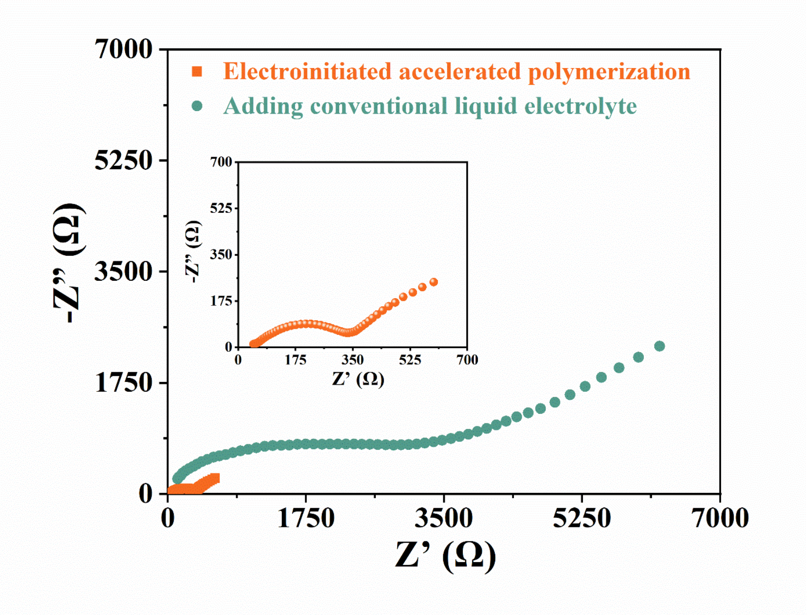

Supplementary Figure 47. EIS Nyquist plots of SSNMBs with NVP electrode and NZZSPO after adding conventional liquid electrolytes or optimized by EAP strategy.

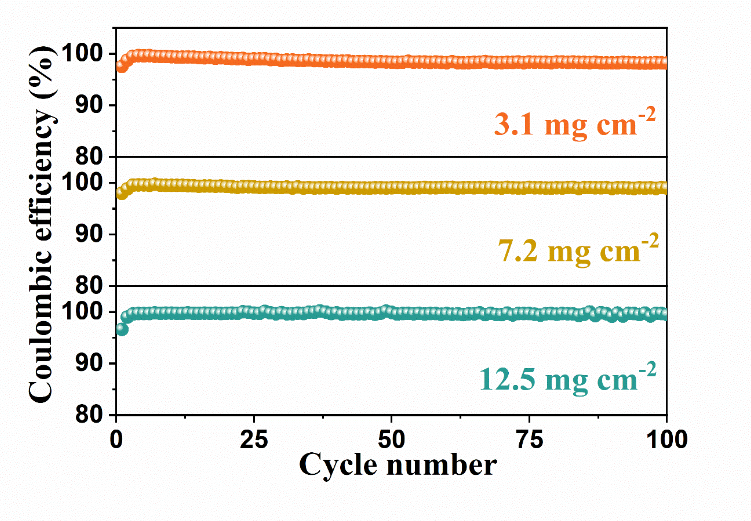

Supplementary Figure 48. Coulombic efficiency with different active material loadings at 0.5 C.

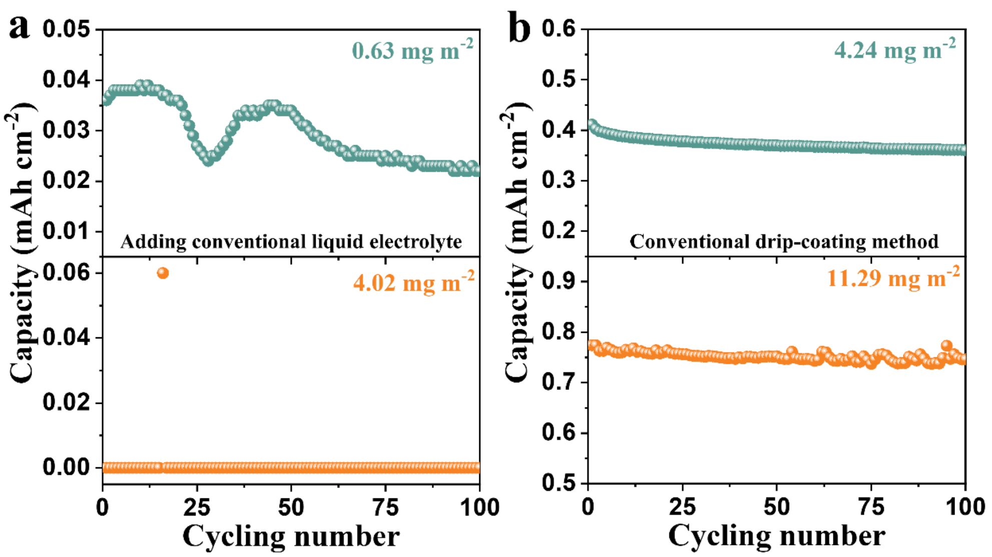

Supplementary Figure 49. Cycling performance of SSNMBs paired with NVP electrode optimized by **a** adding CLE and **b** conventional drip-coating method with different active material loadings at 0.5 C.

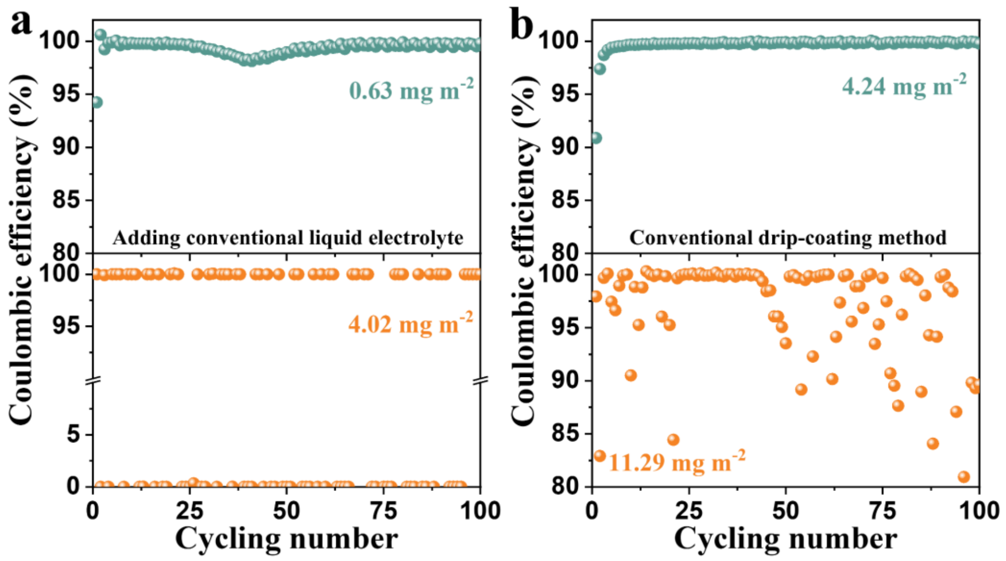

Supplementary Figure 50. Corresponding Coulombic efficiency of SSNMBs optimized by **a** adding CLE and **b** conventional drip-coating method with different active material loadings at 0.5 C.

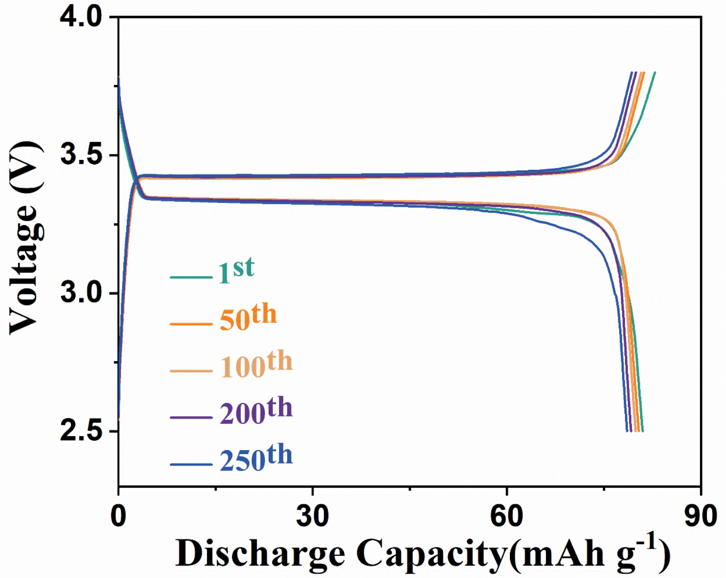

Supplementary Figure 51. Corresponding discharging/charging curves at 0.1 C of Na|NZZSPO|NVP solid-state laminated pouch cell optimized by EAP strategy.

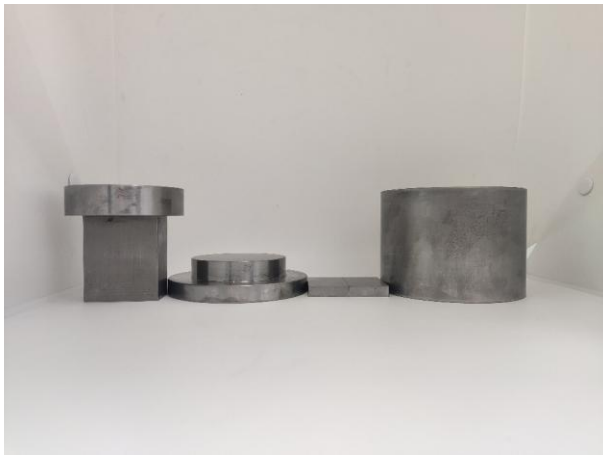

Supplementary Figure 52. Schematic diagram of the homemade tableting mold for solid-state pouch cells.

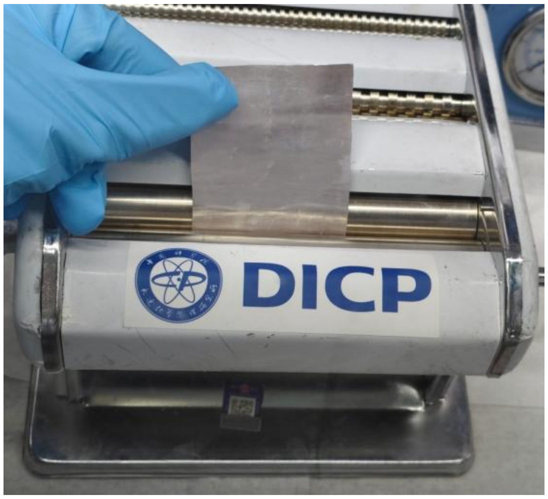

Supplementary Figure 53. Production of Na foil for Na metal solid-state pouch cell by the homemade rolling machine.

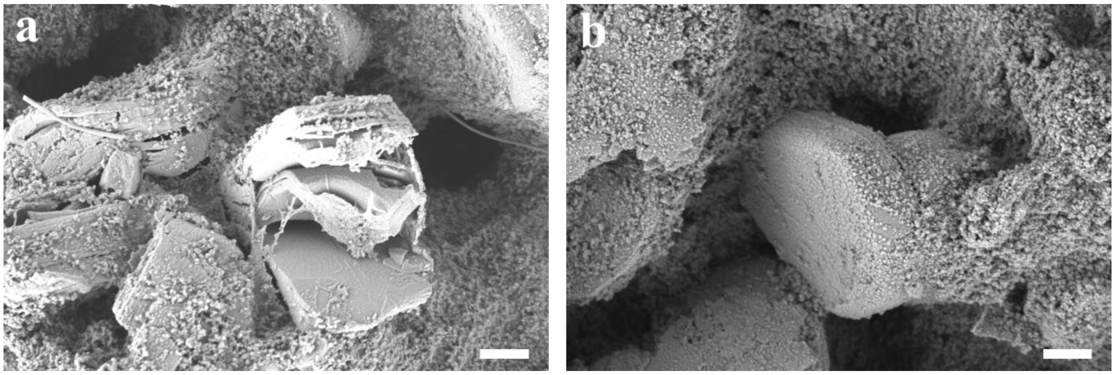

Supplementary Figure 54. SEM images of NFM electrodes for SSNMBs **a** optimized by adding CLE and **b** using EAP strategy after 50 cycles at 0.2 C. Scale bars, 1 μm.

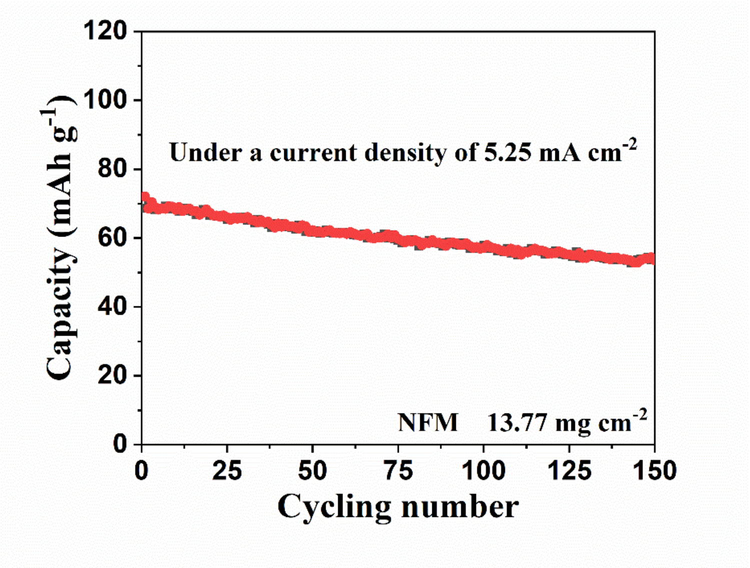

Supplementary Figure 55. Cycling performance of SSNMBs paired with NFM electrode optimized by EAP strategy under a current density of 5.25 mA cm^{-2}.

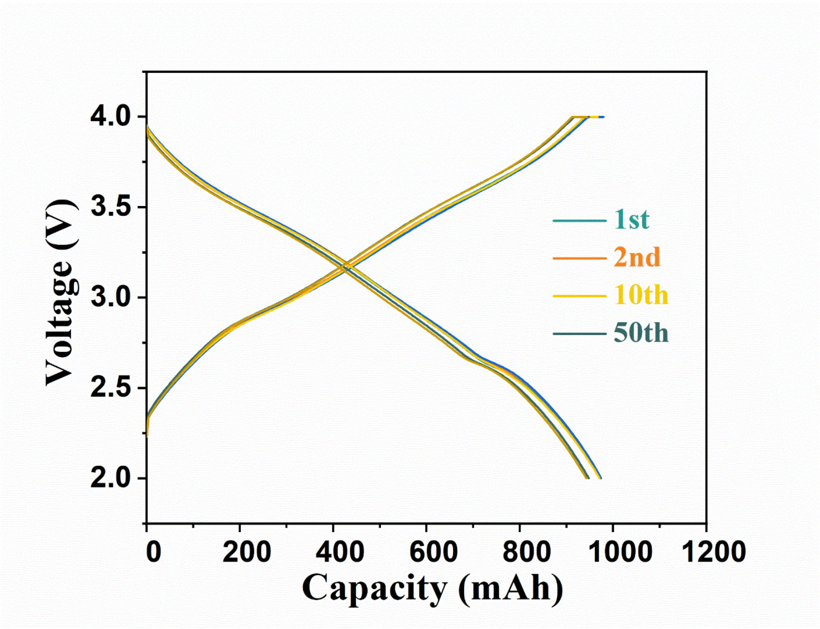

Supplementary Figure 56. Corresponding discharging/charging curves at 0.1 C of Na|NZZSPO|NFM solid-state laminated pouch cell optimized by EAP strategy.

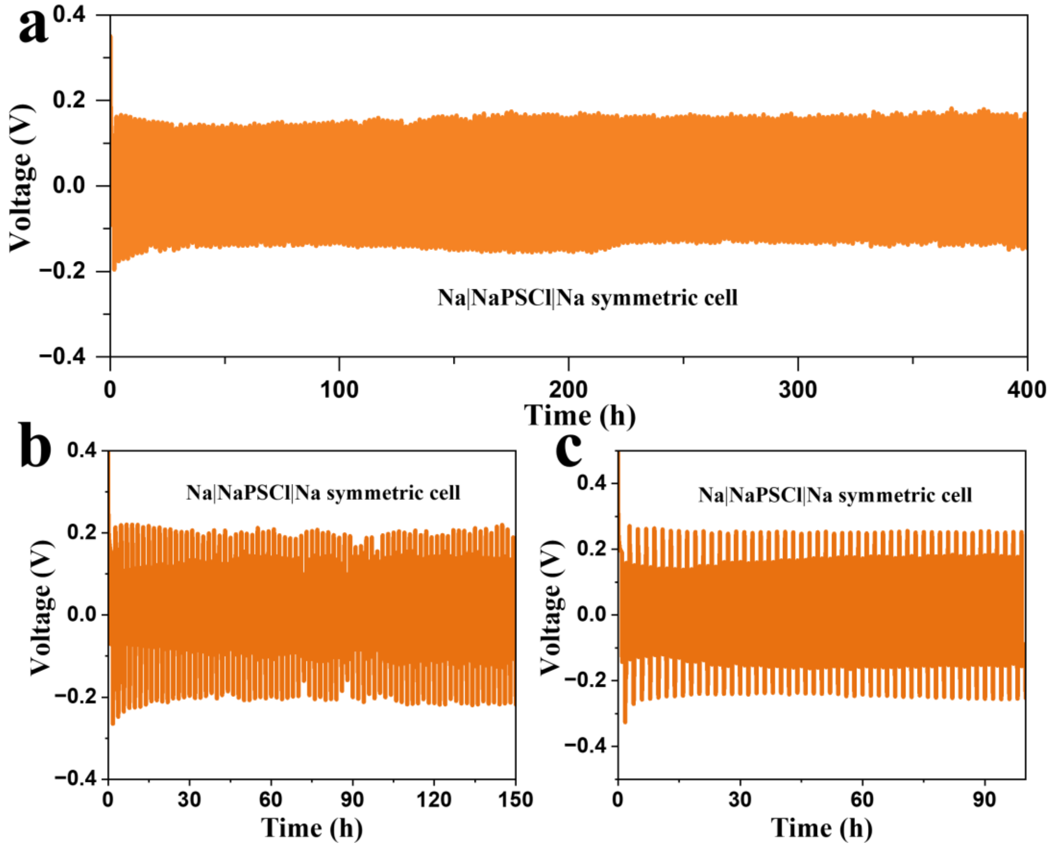

Supplementary Figure 57. Voltage-time profiles of the Na metal-based solid-state symmetric cells with NaPSCl-based sulfide solid electrolyte using EAP healing strategy at the current density of **a** 0.1, **b** 0.5, and **c** 1.0 mA cm^{-2} with the corresponding area capacity of 0.1, 0.5, and 1.0 mAh cm^{-2}.

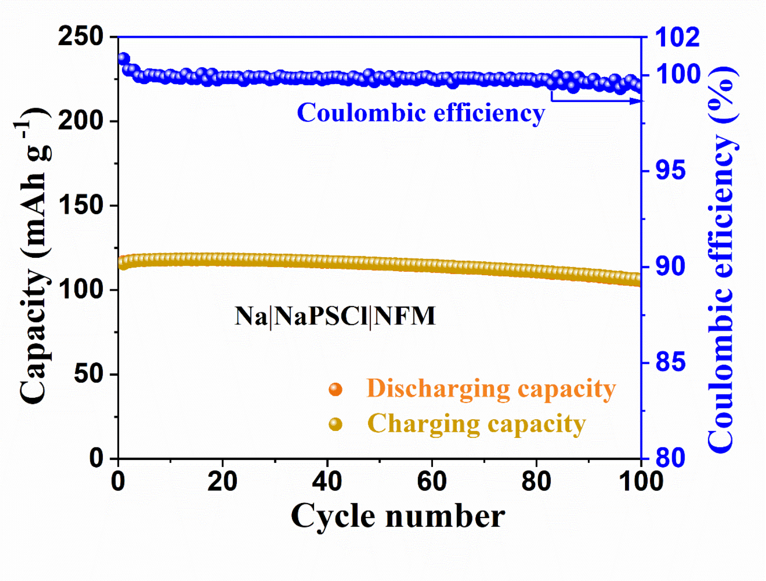

Supplementary Figure 58. Cycling stability of SSNMBs paired with NFM electrode and NaPSCl-based sulfide solid electrolyte at 0.2 C, where the interface is optimized by EAP strategy.

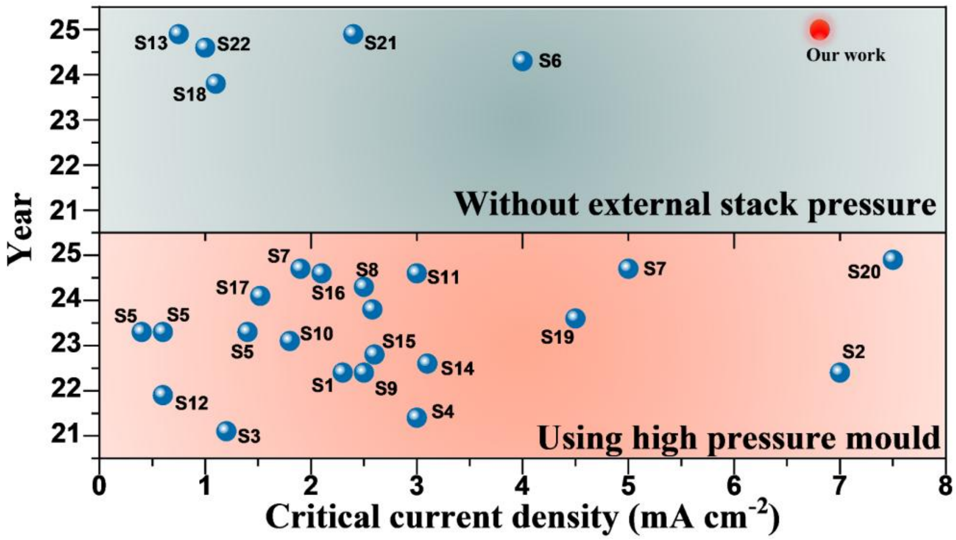

Supplementary Figure 59. Comparison of the reported largest critical current density value of various inorganic solid-state electrolytes using the high-pressure mold and without external stack pressure.

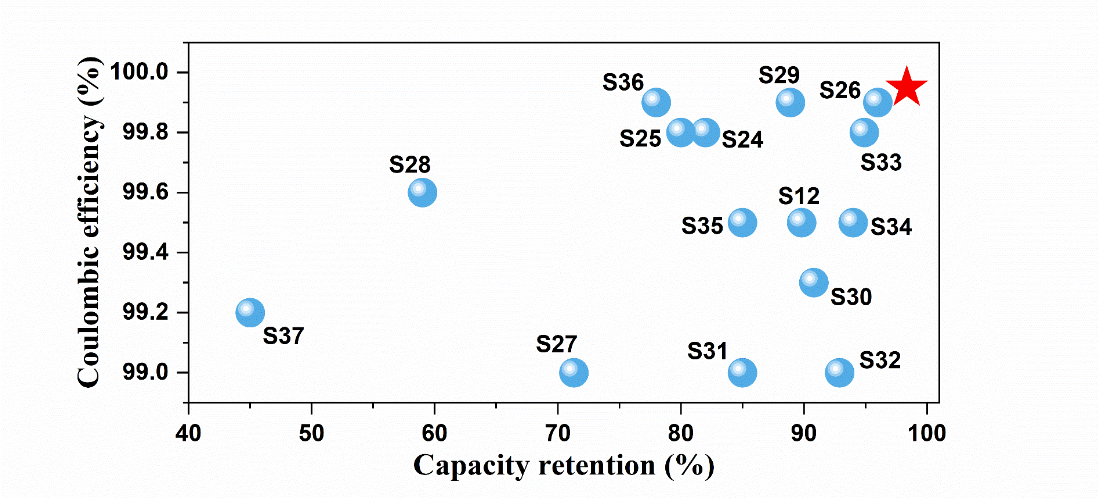

Supplementary Figure 60. Comparison of the reported largest capacity retention and Coulombic efficiency of the reported works within the last 5 years.

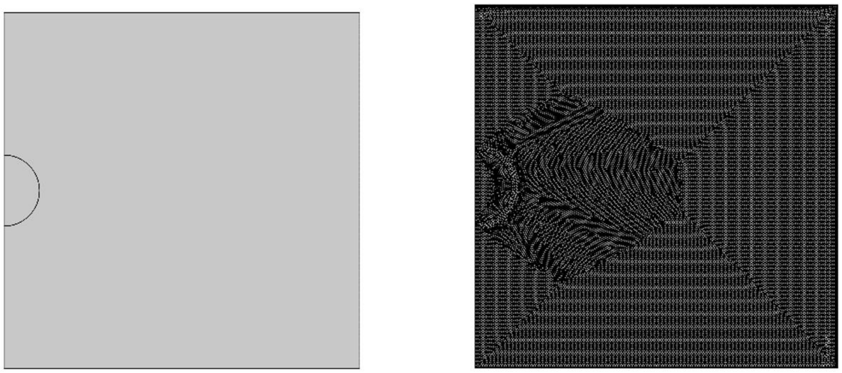

Supplementary Figure 61. Detailed structural models for finite element simulations.

Supplementary Table 1. ICP results for ceramic electrolyte materials with stoichiometry ratio.

Element	Na	Zr	Zn	Si	P
Molar ratio	3.39	1.89	0.11	2.19	0.81

Supplementary Table 2. ICP results for sulfide solid electrolyte materials with stoichiometry ratio.

Element	Na	P
Molar ratio	2.9	1.0

Supplementary Table 3. Comparison of the performance for various reported interfacial optimization strategies.

Optimization strategy	External pressure	CCD value	Coulombic efficiency	Rate performance	Pouch cell
Mechano-electrochemical healing[S38]	35 MPa	/	99.40%	5.0 C	/
Ion-conductive polymer layer[S38]	/	1.58 mA cm^{-2}	98.19%	5.0 C	/
Polymer interface coating[S39]	/	1.1 mA cm^{-2}	/	1.0 C	/
Liquid metal gallium[S21]	50 MPa	1.7 mA cm^{-2}	~100%	~1.5 C	/
Na-Au alloy interface layer[S40]	0 MPa	0.8 mA cm^{-2}	95.20%	5.0 C	Single-layer positive electrode
Polymer interlayer[S41]	0 MPa	1.4 mA cm^{-2}	91.80%	2.0 C	/
KF electron-blocking interlayer[S22]	0 MPa	1.0 mA cm^{-2}	~99.99%	2.0 C	/
Lithiophilic layer[S42]	0 MPa	2.4 mA cm^{-2}	99.50%	3.0 C	/
Our EAP strategy	0 MPa	6.8 mA cm^{-2}	99.80%	15.0 C	1.0 Ah, 100 cycles

Supplementary References

S1. Chi, X. *et al.* An electrochemically stable homogeneous glassy electrolyte formed at room temperature for all-solid-state sodium batteries. *Nat. Commun.* **13,** 285 (2022).

S2. Deng, T. *et al.* Interfacial-engineering-enabled practical low-temperature sodium metal battery. *Nat. Nanotechnol.* **17,** 269-277 (2022).

S3. Huo, H. *et al.* A flexible electron-blocking interfacial shield for dendrite-free solid lithium metal batteries. *Nat. Commun.* **12,** 176 (2021).

S4. Park, R. J.-Y. *et al.* Semi-solid alkali metal electrodes enabling high critical current densities in solid electrolyte batteries. *Nat. Energy* **6,** 314-322 (2021).

S5. Wan, H. *et al.* Critical interphase overpotential as a lithium dendrite-suppression criterion for all-solid-state lithium battery design. *Nat. Energy* **8,** 473-481 (2023).

S6. Zhu, J. *et al.* Long-cycling and High-voltage Solid State Lithium Metal Batteries Enabled by Fluorinated and Crosslinked Polyether Electrolytes. *Angew. Chem. Int. Ed. Engl.* **63,** e202400303 (2024).

S7. Xiong, B.-Q. *et al.* Long-Life All-Solid-State Batteries Enabled by Cold-Pressed Garnet Composite Electrolytes with Enhanced Li^+ Conduction. *Angew. Chem. Int. Ed. Engl.* **137,** e202413502 (2025).

S8. Qiu, P. *et al.* A High-Rate and Long-Life Sodium Metal Battery Based on a $NaB_3H_8 \cdot xNH_3$@NaB_3H_8 Composite Solid-State Electrolyte. *Angew. Chem. Int. Ed. Engl.* **63,** e202401480 (2024).

S9. Shi, P. *et al.* A dielectric electrolyte composite with high lithium-ion conductivity for high-voltage solid-state lithium metal batteries. *Nat. Nanotechnol.* **18,** 602-610 (2023).

S10. Biao, J. *et al.* Inhibiting Formation and Reduction of Li_2CO_3 to LiC_x at Grain Boundaries in Garnet Electrolytes to Prevent Li Penetration. *Adv. Mater.* **35,** e2208951 (2023).

S11. Jin, Y. *et al.* Fluorinated $Li_{10}GeP_2S_{12}$ Enables Stable All-Solid-State Lithium Batteries. *Adv. Mater.* **35,** e2211047 (2023).

S12. Wang, X., Chen, J., Wang, D. & Mao, Z. Improving the alkali metal electrode/inorganic solid electrolyte contact via room-temperature ultrasound solid welding. *Nat. Commun.* **12,** 7109 (2021).

S13. Yang, S. *et al.* Imaging dendrite growth in solid-state sodium batteries using fluorescence tomography technology. *Sci. Adv.* **10,** eadr0676 (2024).

S14. Lee, S. *et al.* Design of a lithiophilic and electron-blocking interlayer for dendrite-free lithium-metal solid-state batteries. *Sci. Adv.* **8,** eabq0153 (2022).

S15. Feng, W. *et al.* Stabilization of garnet/Li interphase by diluting the electronic conductor. *Sci. Adv.* **8,** eadd8972 (2022).

S16. Zhang, N. *et al.* Homogeneous Fluorine Doping toward Highly Conductive and Stable $Li_{10}GeP_2S_{12}$ Solid Electrolyte for All-Solid-State Lithium Batteries. *Adv. Mater.* **36,** e2408903 (2024).

S17. Xu, S. *et al.* Performance Enhancement of the Li_6PS_5Cl-Based Solid-State Batteries by Scavenging Lithium Dendrites with $LaCl_3$-Based Electrolyte. *Adv. Mater.* **36,** e2310356 (2024).

S18. Ni, Q. *et al.* Piezoelectric Interlayer Enabling a Rechargeable Quasisolid-State Sodium Battery at 0 °C. *Adv. Mater.* **36,** e2309298 (2024).

S19. Guo, Y. *et al.* Fluorinating All Interfaces Enables Super-Stable Solid-State Lithium Batteries by In Situ Conversion of Detrimental Surface Li_2CO_3. *Adv. Mater.* **36,** e2308493 (2024).

S20. Li, W. *et al.* Superionic conducting vacancy-rich β-Li_3N electrolyte for stable cycling of all-

solid-state lithium metal batteries. *Nat. Nanotechnol.* **20,** 265-275 (2025).
S21. Ji, W. *et al.* Interface engineering enabling thin lithium metal electrodes down to 0.78 μm for garnet-type solid-state batteries. *Nat. Commun.* **15,** 9920 (2024).
S22. Zhang, C. *et al.* An electron-blocking interface for garnet-based quasi-solid-state lithium-metal batteries to improve lifespan. *Nat. Commun.* **15,** 5325 (2024).
S23. Zhu, F. *et al.* Trace Fluorinated Carbon Dots Driven Li-Garnet Solid-State Batteries. *Angew. Chem. Int. Ed. Engl.* **63,** e202410016 (2024).
S24. Cao, D. *et al.* Amphipathic Binder Integrating Ultrathin and Highly Ion-Conductive Sulfide Membrane for Cell-Level High-Energy-Density All-Solid-State Batteries. *Adv. Mater.* **33,** e2105505 (2021).
S25. Chen, J. *et al.* Multiple Dynamic Bonds-Driven Integrated Cathode/Polymer Electrolyte for Stable All-Solid-State Lithium Metal Batteries. *Angew. Chem. Int. Ed. Engl.* **62,** e202307255 (2023).
S26. Zhu, J. *et al.* A Multilayer Ceramic Electrolyte for All-Solid-State Li Batteries. *Angew. Chem. Int. Ed. Engl.* **60,** 3781-3790 (2021).
S27. Wu, K. *et al.* SnF2-Catalyzed Lithiophilic-Lithiophobic Gradient Interface for High-Rate PEO-Based All-Solid-State Batteries. *Angew. Chem. Int. Ed. Engl.* **63,** e202410347 (2024).
S28. Wang, X. *et al.* Hybrid Electrolyte with Dual-Anion-Aggregated Solvation Sheath for Stabilizing High-Voltage Lithium-Metal Batteries. *Adv. Mater.* **33,** e2007945 (2021).
S29. Sung, J. *et al.* Ultra-Thin Lithium Silicide Interlayer for Solid-State Lithium-Metal Batteries. *Adv. Mater.* **35,** e2210835 (2023).
S30. Su, H. *et al.* A scalable Li-Al-Cl stratified structure for stable all-solid-state lithium metal batteries. *Nat. Commun.* **15,** 4202 (2024).
S31. Sheng, O. *et al.* In Situ Construction of a LiF-Enriched Interface for Stable All-Solid-State Batteries and its Origin Revealed by Cryo-TEM. *Adv. Mater.* **32,** e2000223 (2020).
S32. Ren, X. *et al.* Designing Advanced In Situ Electrode/Electrolyte Interphases for Wide Temperature Operation of 4.5 V Li||$LiCoO_2$ Batteries. *Adv. Mater.* **32,** e2004898 (2020).
S33. Mu, K. *et al.* Hybrid Crosslinked Solid Polymer Electrolyte via In-Situ Solidification Enables High-Performance Solid-State Lithium Metal Batteries. *Adv. Mater.* **35,** e2304686 (2023).
S34. Luo, D. *et al.* Constructing multifunctional solid electrolyte interface via in-situ polymerization for dendrite-free and low N/P ratio lithium metal batteries. *Nat. Commun.* **12,** 186 (2021).
S35. Liu, S. *et al.* Solid-State Lithium Metal Batteries with Extended Cycling Enabled by Dynamic Adaptive Solid-State Interfaces. *Adv. Mater.* **33,** e2008084 (2021).
S36. Lin, X. *et al.* A family of dual-anion-based sodium superionic conductors for all-solid-state sodium-ion batteries. *Nat. Mater.* **24,** 83-91 (2025).
S37. He, F., Tang, W., Zhang, X., Deng, L. & Luo, J. High Energy Density Solid State Lithium Metal Batteries Enabled by Sub-5 μm Solid Polymer Electrolytes. *Adv. Mater.* **33,** e2105329 (2021).
S38. Lee, S. et al. Mechano-Electrochemical Healing at the Interphase Between LiNi0.8Co0.1Mn0.1O2 and Li6PS5Cl in All-Solid-State Batteries. Adv. Energy Mater. 2405782 (2025).
S39. Yang, G. *et al.* A Bridge between Ceramics Electrolyte and Interface Layer to Fast Li^+ Transfer for Low Interface Impedance Solid-State Batteries. *Adv. Funct. Mater.* **33,** 2211387 (2023).
S40. Li, D. *et al.* Atomically bonding Na anodes with metallized ceramic electrolytes by ultrasound

welding for high-energy/power solid-state sodium metal batteries. *Carbon Energy* **5,** e299, (2023).

S41. Miao, X. *et al.* Isotropous Sulfurized Polyacrylonitrile Interlayer with Homogeneous Na^+ Flux Dynamics for Solid-State Na Metal Batteries. *Adv. Energy Mater.* **11,** 2003469 (2021).

S42. Meng, L., Zhang, Y., Zhou, X., Lei, M. & Li, C. Li_2CO_3-affiliative mechanism for air-accessible interface engineering of garnet electrolyte via facile liquid metal painting. *Nat. Commun*. **11**, 3716 (2020).
