# Supplementary material for: Electroinitiated interfacial healing for external pressure-free solid-state sodium metal batteries
Source: Nat Commun. 2025 Oct 30;16:9613. doi: 10.1038/s41467-025-64612-7 (PMC12575646; doi:10.1038/s41467-025-64612-7)
Supplement: Supplementary file 2 — Description of Additional Supplementary Files [file 41467_2025_64612_MOESM2_ESM.pdf]

Description of Additional Supplementary Files

Supplementary Data 1: Configurations for various molecular dynamics trajectories and optimized molecular structure.

Supplementary Movie 1. Simulated coating process by finite element simulation through conventional drip-coating method (left) and our electroinitiated accelerated polymerization strategy (right).

Supplementary Movie 2. Simulated flow process of our electroinitiated accelerated polymerization strategy by finite element simulation.

Supplementary Movie 3. Crack healing process using our electroinitiated accelerated polymerization strategy taken with microscopy.

Supplementary Movie 4. In situ optical microscopy observations of oxide solid electrolytes modified by adding conventional liquid electrolyte.

Supplementary Movie 5. In situ optical microscopy observations of oxide solid electrolytes modified by our electroinitiated accelerated polymerization strategy.
